# Lignin nanoparticles in food packaging: a sustainable approach to material design and performance enhancement

**DOI:** 10.3389/fnut.2025.1659877

**Published:** 2025-11-28

**Authors:** Jinqian Peng, Yu Wang

**Affiliations:** 1 School of Business Administration, The Hong Kong Polytechnic University, Hong Kong, China; 2 Division of Biostatistics, Medical College of Wisconsin, Milwaukee, WI, United States

**Keywords:** lignin nanoparticles, polysaccharide-based films, food packaging, nanocomposites, food safety

## Abstract

The global food packaging industry is undergoing a transformative shift toward sustainable alternatives to conventional petroleum-based materials, driven by escalating environmental concerns, regulatory pressures, and consumer demand for greener products. Biodegradable polymers such as chitosan, starch, cellulose, PLA, and PBS have gained prominence due to their renewability and reduced ecological footprint. However, their inherent limitations, including poor mechanical strength, moisture sensitivity, and limited bioactivity, have restricted widespread adoption. Lignin nanoparticles (LNPs), derived from the abundant and underutilized biopolymer lignin, have emerged as multifunctional nanofillers capable of significantly enhancing the structural and functional properties of biodegradable films. Owing to their antioxidant, UV-blocking, antimicrobial, and barrier-enhancing characteristics, LNPs improve film performance while aligning with circular economy principles. This review critically examines the sources, structural characteristics, extraction methods, and synthesis techniques of lignin, emphasizing their impact on nanoparticle formation and functionality. It further explores recent advances in LNP-reinforced packaging systems across diverse biopolymer matrices, including polysaccharides, proteins, and biodegradable polyesters. Special attention is given to interfacial interactions, dispersion behavior, and structure–property correlations. Finally, emerging trends such as LNP surface engineering, smart coating systems, and hybrid fabrication strategies are discussed in the context of future scalability, recyclability, and regulatory compliance.

## 1 Introduction

The global food industry is increasingly challenged by the urgent need to transition toward more sustainable packaging solutions, driven by escalating concerns over environmental degradation, resource depletion, and human health risks associated with conventional materials (1). Petroleum-derived plastics, aluminum, and perfluorinated compounds have been the mainstay of food packaging due to their excellent barrier properties, mechanical strength, and low cost (2). However, these materials present significant environmental and sustainability drawbacks. Petroleum-based plastics, including polyethylene (PE), polypropylene (PP), and polyethylene terephthalate (PET), are non-biodegradable and widely used for their flexibility and barrier capabilities (3–5), yet their persistence in ecosystems leads to accumulation in landfills and marine environments, contributing to global microplastic pollution (6). The global production of plastics surpassed 400 million tons in 2022, with food packaging accounting for approximately 40% of total consumption (7). Alarmingly, less than 20% of plastics are effectively recycled, exacerbating long-term waste management challenges (8). Although aluminum is technically recyclable and commonly used in food packaging as metalized films or containers, its production from bauxite ore involves highly energy-intensive processes that result in considerable greenhouse gas emissions and ecological disruption through land degradation and mining waste (9). Moreover, the use of metallic films in food packaging raises concerns about material circularity and environmental compatibility, particularly in applications where recovery and recycling are impractical or uneconomical. In contrast, biodegradable packaging materials can be composted or decomposed in natural environments, avoiding such resource-intensive end-of-life scenarios. Perfluorinated organic compounds (PFCs), often used to impart grease and water resistance to packaging, have also been linked to endocrine disruption, bioaccumulation, and long-term ecological toxicity. The detection of PFC residues in food products and human serum samples has further intensified public health concerns (10, 11), reinforcing the urgent need to develop safer, functional, and environmentally benign alternatives such as biodegradable films containing lignin nanocomposites.

These challenges have accelerated research into biodegradable packaging materials derived from renewable resources. Among biopolymers, polysaccharides (e.g., starch, cellulose), proteins (e.g., gelatin, soy protein), and polyesters (e.g., polylactic acid, polyhydroxyalkanoates) are prominent candidates. Starch-based films offer biodegradability and film-forming abilities but suffer from poor water vapor resistance, brittleness, and mechanical weakness (12, 13). Cellulose, abundant and renewable, provides good mechanical strength and oxygen barrier properties but remains hydrophilic, limiting its performance under high humidity conditions (14). Furthermore, the native crystallinity of cellulose often necessitates chemical modifications or blending with other biopolymers to tailor its functional properties (15, 16). Chitosan films are inherently antimicrobial and biodegradable but present processing challenges, high water sensitivity, and brittleness under dry conditions (17). Proteins such as gelatin and soy protein have been widely investigated for packaging films but face issues of poor mechanical stability and moisture sensitivity (18). Thus, reinforcing these biodegradable polymers with functional additives, nanoparticles, or cross-linkers has emerged as a promising strategy to overcome their intrinsic limitations.

Lignin, the second most abundant biopolymer after cellulose, has received growing attention in this context. Accounting for up to 30% of lignocellulosic biomass, lignin is a complex, three-dimensional aromatic macromolecule characterized by high phenolic content, crosslinked structures, and inherent antioxidative and antimicrobial properties (19, 20). The unique chemical structure of lignin, composed of p-hydroxyphenyl (H), guaiacyl (G), and syringyl (S) units, imparts hydrophobicity and stability, making it a suitable candidate for enhancing barrier and functional properties of biodegradable polymer films (21, 22). Despite its abundance—estimated at over 70 million tons annually as an industrial byproduct—lignin remains underutilized, primarily being incinerated for low-value energy recovery in pulp and paper mills. Its underexploitation is attributed to the heterogeneity in lignin’s structure depending on the botanical source and extraction method, which complicates its direct utilization in high-value applications.

The valorization of lignin into value-added products, such as functional fillers or antioxidants, aligns well with circular economy strategies, aiming to reduce waste and maximize resource efficiency (23). Its functional groups, such as hydroxyl, methoxyl, and carboxyl groups, offer opportunities for chemical modification, improving its compatibility with various biopolymer matrices (24).

Recent advancements in nanotechnology have enabled the production of lignin nanoparticles (LNPs), which dramatically enhance the applicability of lignin in food packaging. LNPs, typically ranging between 50 and 300 nm, exhibit high surface area-to-volume ratios, improved dispersibility, and enhanced functional reactivity compared to native lignin (25). LNPs are emerging as a versatile and sustainable material with promising applications across various industries. The global lignin market has seen significant growth, with kraft lignin production increasing by 150% from 2014 to 2018 (26). These nanoparticles offer advantages such as non-toxicity, low cost, and potential biodegradability, making them attractive for use in antioxidants, UV-protectants, drug carriers, and tissue engineering (27). The world market for nanoparticles is expected to increase markedly in the coming years, suggesting a growing demand for lignin nanoparticles. However, challenges remain in scaling up technologies and optimizing lignin applications to achieve technical and economic feasibility (26). A critical review by Österberg et al. (28) highlights the sustainability, performance, stability, and degradation aspects of spherical lignin particles, further emphasizing their potential in various applications (28).

Preparation methods for LNPs include solvent–antisolvent precipitation, ultrasonication, aerosol flow reactors, and dialysis, each influencing the particle morphology, size distribution, and surface chemistry (29). Antisolvent precipitation is one of the most commonly employed approaches, yielding spherical and uniform LNPs through rapid aggregation of lignin in poor solvents such as water or acidified ethanol. This method is simple, scalable, and free from harsh reagents, although it may require process optimization to avoid particle aggregation (30). Alternatively, solvent exchange (dialysis) offers a greener route for producing highly stable and pure nanoparticles by gradually replacing organic solvents with water, allowing controlled self-assembly of lignin into nanoscale structures. Although time-consuming and relatively costly due to membrane use, this approach ensures high colloidal stability and avoids post-synthesis aggregation (31). Ultrasonication provides a rapid and chemical-free method by utilizing acoustic cavitation to break down bulk lignin into nanoparticles. While easy to implement, it may lead to broader size distributions and potential oxidative modification of lignin unless conditions are carefully controlled (32). For continuous and scalable production, aerosol flow reactor synthesis has emerged as a promising method where lignin droplets are atomized and thermally dried to yield dry, uniform LNPs powders suitable for large-scale packaging applications (33).

Controlling the particle size and surface charge is crucial, as these parameters affect the stability of the nanoparticles and their interaction with the biodegradable polymer matrix. Incorporating LNPs into biodegradable films has demonstrated substantial improvements in mechanical properties, barrier functions, and active packaging functionalities. Studies have shown that LNPs enhance tensile strength, Young’s modulus, and elasticity while reducing water vapor permeability and oxygen transmission rates (34). Furthermore, LNPs act as UV-blocking agents, protecting packaged foods from photooxidation and extending shelf life (35). The antioxidant activity of LNPs helps to retard lipid oxidation in packaged foods, while their antimicrobial properties inhibit bacterial and fungal growth, reducing spoilage and enhancing food safety without relying on synthetic additives (19, 36). Such multifunctional benefits position LNPs as attractive candidates for developing active and intelligent packaging systems.

Despite these promising findings, challenges persist regarding the scalability of LNPs production, reproducibility of particle characteristics, potential migration of lignin-derived compounds into food, and regulatory hurdles. Variations in lignin source (kraft, organosolv, soda), extraction methods, and particle preparation techniques influence the physicochemical properties and performance of LNPs-reinforced biodegradable films (37, 38). Additionally, aggregation of LNPs at high loading concentrations can compromise film uniformity and mechanical performance, necessitating strategies such as surface functionalization or compatibilizer addition (39). Regulatory approval remains a major hurdle, as detailed toxicological assessments and migration studies are required to meet food contact material safety standards set by authorities such as the U. S. Food and Drug Administration (FDA) and the European Food Safety Authority (EFSA) (40). From a sustainability perspective, integrating lignin valorization into biodegradable packaging systems not only offers material performance benefits but also contributes to the broader goals of reducing industrial waste streams and creating high-value, biodegradable materials (41). Life cycle assessment (LCA) studies are beginning to show that lignin-enhanced biodegradable films have lower overall environmental impacts compared to traditional petrochemical-based packaging (42).

It is important to emphasize that the scope of this review centers on biodegradable food packaging materials incorporating LNPs, irrespective of whether the matrix polymer is bio-based or synthetic in origin. While many of the matrices discussed, such as starch, chitosan, and cellulose, are derived from renewable resources, others like polyvinyl alcohol (PVA) originate from petrochemical sources but are still biodegradable under industrial conditions. Such materials serve as transitional or benchmark systems in sustainable packaging development and offer valuable insight into structure–function relationships of LNP-based composites. As such, this review considers both natural and synthetic biodegradable matrices to provide a comprehensive understanding of LNP integration strategies and performance outcomes. This review critically explores the incorporation of lignin nanoparticles into biodegradable films for food packaging applications. It discusses the current state of knowledge regarding LNPs synthesis, characterization, and integration into different biodegradable polymer matrices. The impact of LNPs on the physicochemical, mechanical, and functional properties of composite films is analyzed, alongside the existing challenges and perspectives for future research and commercialization. By elucidating these aspects, the review aims to foster the development of next-generation sustainable food packaging materials aligned with circular economy principles and emerging consumer expectations for greener packaging solutions.

## 2 Source and structure of lignin

**Figure 1:**
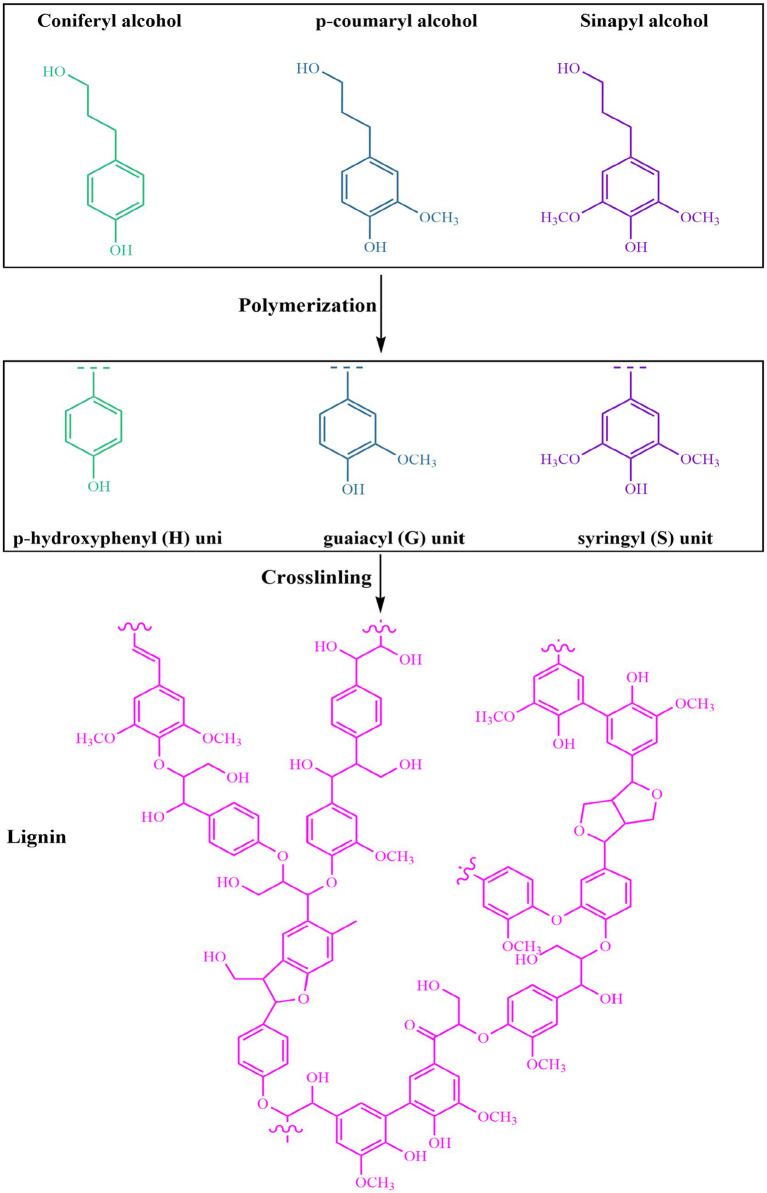
Schematic representation of lignin biosynthesis from monolignol precursors. The three main monolignols, p-coumaryl alcohol, coniferyl alcohol, and sinapyl alcohol, polymerize via radical coupling to form the structural units of lignin: p-hydroxyphenyl (H), guaiacyl (G), and syringyl (S) units, respectively. These units are then crosslinked into a complex and highly branched lignin macromolecule.

Lignin is an irregular, three-dimensional aromatic polymer found abundantly in lignocellulosic biomass. Its content typically ranges from 15 to 30% of the plant’s dry mass, depending on the species and the specific tissue type (20, 43). In addition to cellulose and hemicellulose, lignin is essential for providing plants with structural support, water resistance, and protection against microbial attacks (44). Its complex architecture and wide range of natural sources largely determine its physical and chemical properties, making lignin an increasingly valuable material for developing sustainable products such as biodegradable food packaging (45). Lignin is formed through an oxidative radical coupling process involving three key monolignol precursors: p-coumaryl alcohol, coniferyl alcohol, and sinapyl alcohol (46). These compounds, which differ mainly in the number of methoxy groups attached to their aromatic structures, polymerize to produce p-hydroxyphenyl (H), guaiacyl (G), and syringyl (S) units, respectively (Figure 1). The combination and arrangement of these structural units give lignin its highly complex and irregular macromolecular framework (47). The relative abundance of H, G, and S units depends strongly on the plant source. In softwoods, lignin is mainly built from guaiacyl (G) units, making up about 90–95% of its structure. In contrast, hardwood lignins contain a blend of guaiacyl and syringyl units, with syringyl groups generally being more abundant, representing between 50 and 75%. Meanwhile, lignins isolated from grasses and herbaceous species are notable for their higher levels of hydroxyphenyl (H) units, typically ranging from 10 to 25%, while also containing significant amounts of G and S units (43, 48, 49).

The high structural complexity of lignin results not only from its diverse monomer composition but also from the variety of chemical bonds formed during its polymerization. Among these, *β*-O-4 (β-aryl ether) linkages are the most prevalent, accounting for approximately 43–65% of all interunit connections, especially in lignin found in native plant cell walls (49). Besides *β*-O-4 bonds, lignin contains other important linkages such as *β*-5 (phenylcoumaran), 5–5 (biphenyl), β-β (resinol), and 4-O-5 (diaryl ether), each contributing differently to its three-dimensional structure and overall stability (50). The β-O-4 bonds, due to their chemical nature, are relatively easy to break under appropriate conditions, making them a primary target in depolymerization and valorization processes (51). In contrast, carbon–carbon linkages like *β*-5 and 5–5 are much stronger and more resistant to cleavage, which explains the notable thermal stability and chemical durability of lignin polymers (52). The macromolecular architecture of lignin is highly irregular and extensively branched, lacking the uniformity and repeating structures typically seen in polysaccharides like cellulose. Because of its random polymerization during biosynthesis, many researchers suggest that lignin should be described as a random polyphenolic copolymer rather than a traditional biopolymer (53). In its native form, lignin is not present as a completely isolated molecule; instead, it is closely bonded to hemicelluloses through covalent linkages, resulting in the formation of lignin–carbohydrate complexes (LCCs). These associations significantly impact the extraction, structural characteristics, and functional behavior of lignin (54).

The molecular weight of lignin shows considerable variation, largely depending on the plant source and several biological factors. In general, native lignin has a weight-average molecular weight (Mw) that falls between 3,000 and 50,000 g/mol, although in some cases, these values can differ significantly based on species type and the conditions under which the lignin is isolated (55). Lignins derived from softwood species usually possess higher Mw values and broader polydispersity indexes when compared to those extracted from hardwoods and grasses, indicating a greater degree of branching and molecular complexity (56, 57). Differences in molecular size directly influence key properties of lignin, such as its solubility, thermal stability, and chemical reactivity, all of which are critical for its modification and use in the development of advanced composite materials (58). The chemical properties of lignin are largely determined by the wide variety of functional groups present within its structure. Among these, phenolic hydroxyl groups, mostly found on non-condensed aromatic rings, play a vital role in providing antioxidant capabilities, radical scavenging efficiency, and the potential for crosslinking reactions (59). Methoxy groups, located at the aromatic rings, significantly affect the electron distribution and redox behavior of lignin, which in turn influences its ability to absorb ultraviolet light and stabilize radicals (60). Aliphatic hydroxyl groups, attached to the lignin side chains, enhance its polarity and offer sites for chemical transformations such as esterification (61). Additionally, carboxyl and carbonyl groups, often introduced through oxidation reactions, provide more reactive centers and increase the hydrophilic character of lignin molecules (62).

The botanical source plays a crucial role in determining the detailed structure of lignin. In softwood species, lignin is mainly composed of guaiacyl (G) units, resulting in a polymer that is densely crosslinked due to a high frequency of *β*-5 and 5–5 carbon–carbon bonds, which contributes to its notable thermal stability and condensed nature (63). In contrast, hardwood lignins contain a greater proportion of syringyl (S) units, which promote a more linear molecular structure dominated by *β*-O-4 linkages, making them generally more reactive and easier to modify chemically (64). Lignins from grasses present an even more complex architecture, as they incorporate significant amounts of hydroxyphenyl (H) units and unique cross-linkages involving p-coumarates and ferulates, which are less commonly found in lignins from woody plants (65). Thus, the behavior and properties of lignin are largely determined by its unique combination of monolignol composition, the variety of interunit linkages, molecular weight characteristics, and abundance of functional groups. Gaining a detailed understanding of these structural features is essential for optimizing lignin’s role in the development of innovative materials. This knowledge is particularly important in the growing area of biodegradable-based food packaging, where lignin’s antioxidant, antimicrobial, and ultraviolet (UV) protective abilities are increasingly being recognized and utilized.

## 3 Lignin extraction methods: classification and perspectives

The extraction of lignin from lignocellulosic biomass is a critical step in biomass valorization, offering pathways for the development of bio-based chemicals, materials, and fuels. Over the past decades, a wide range of lignin extraction techniques have been developed, encompassing traditional chemical methods, organic solvent-based approaches, physical and green pretreatments, biological methods, and specialized extraction strategies. Each technique differs in its mechanism of action, the structural characteristics of the recovered lignin, and its associated advantages and limitations. Table 1 provides a comparative overview of these major lignin extraction technologies, summarizing their underlying mechanisms, lignin characteristics, process advantages, and challenges. This section systematically discusses the principles, operational features, and perspectives of key lignin extraction methods, highlighting both conventional and emerging strategies.

**Table 1:** Comparative overview of conventional and emerging lignin extraction technologies.

Extraction technique	Reagent/Condition	Mechanism of action	Lignin characteristics	Advantages	Challenges
Kraft process	Sodium hydroxide + sodium sulfide	Cleavage of β-O-4 bonds by nucleophilic attack and sulfuration	High phenolic hydroxyl groups; sulfur content	Industrial scalability; strong delignification	Sulfur contamination; lower purity
Lignosulfonate process	Sulfite salts or sulfurous acids	Sulfonation and hydrolysis of lignin structures	High sulfonate content; high water solubility	Good dispersibility; commercial applications	High impurity level; structural condensation
Soda process	Sodium hydroxide	Alkaline hydrolysis of β-O-4 and β-5 linkages	Sulfur-free; enriched phenolic structures	High purity; sulfur-free lignin	High condensation; lower structural reactivity
Organosolv process	Organic solvents with or without acid catalyst	Solvolysis and selective ether bond cleavage	Sulfur-free; high purity; hydrophobic	Preservation of native lignin structure; solvent recovery possible	Solvent cost; corrosion issues
Deep eutectic solvents (DES)	Choline chloride-based mixtures	Cleavage of aryl ether and carbon–carbon bonds	Relatively pure; green solvent extracted	Eco-friendly; customizable solvent design	Scale-up complexity; solvent recycling needed
Ionic liquid extraction (ILs)	Imidazolium- or phosphonium-based ILs	Solubilization of lignin via disruption of intermolecular forces	High solubility; clean fractionation	Selective lignin extraction; low emissions	High solvent cost; regeneration difficulties
Supercritical fluid extraction (SCF)	Supercritical CO₂ with or without cosolvents	Physical penetration and chemical cleavage of ether linkages	High purity; minimal ash content	Green technology; tunable extraction	Expensive equipment; operational complexity
Non-thermal plasma (NTP)	Plasma discharge with reactive gases	C–C and C–H bond cleavage; surface oxidation	Aromatic structure largely preserved	Short treatment time; low energy input	Optimization for industrial scaling needed
Microwave-assisted extraction (MAE)	Microwave irradiation with solvents	Rapid internal heating, bond weakening and cleavage	Enhanced extractability; structural diversity	Fast, energy-saving extraction	Limited penetration depth; possible overheating
Enzymatic hydrolysis	Cellulase and hemicellulase enzymes	Removal of polysaccharides; lignin remains insoluble	Native-like lignin structure; low sulfur	Mild eco-friendly conditions	Residual proteins and carbohydrates contamination

### 3.1 Traditional chemical methods

#### 3.1.1 Kraft lignin extraction

Kraft lignin (KL) is a by-product generated during the kraft pulping process, which remains the most widely used industrial method for removing lignin from lignocellulosic biomass, particularly wood sources (66). In this process, biomass is treated with a solution called white liquor, composed of sodium hydroxide and sodium sulfide, at elevated temperatures ranging from 165 to 175 °C under strongly alkaline conditions (pH 13–14) (67). The highly basic environment promotes the ionization of phenolic hydroxyl groups, resulting in the formation of quinone methide intermediates. These reactive species are then targeted by bisulfide ions through nucleophilic attack (67, 68), leading to the cleavage of *β*-O-4-aryl ether bonds — essential linkages that account for approximately 50–70% of the internal structure of lignin (69). Additionally, the process breaks *α*-aryl and α-alkyl ether bonds and introduces small amounts (about 1–3%) of thiol groups into the lignin structure (70).

After delignification, lignin is separated from the black liquor through acidification, often using sulfuric acid, to lower the pH to around 5–7.5 (71). The resulting kraft lignin typically shows a broad molecular weight range, from 500 to 20,000 Da, depending on the type of biomass processed (71). Compared to lignosulfonates, kraft lignin is considered purer, as it contains fewer inorganic components and carbohydrate impurities (72). However, due to its low sulfur content, it has limited water solubility and usually requires chemical modification before practical application (73). Today, kraft lignin is primarily utilized in the production of polymer composites, industrial dispersants, and as a feedstock for the synthesis of biofuels and various value-added chemicals after undergoing further chemical processing or depolymerization (66).

#### 3.1.2 Soda lignin extraction

Soda lignin is obtained by processing biomass—especially herbaceous plants such as wheat straw and bagasse—using sodium hydroxide (NaOH) solutions, without incorporating any sulfur-based chemicals (66). In this method, biomass is typically treated with 13–16 wt% NaOH at elevated temperatures ranging from 140 to 170 °C for about 90 min (66). The strong alkaline environment causes the breakdown of *β*-O-4 and β-5 bonds within the lignin structure, leading to the formation of new vinyl ether and phenolic hydroxyl groups (74). After the cooking stage, the lignin is separated from the black liquor by acidifying the solution to approximately pH 5.5, prompting lignin precipitation (75). One of the major distinctions between soda lignin and kraft lignin is that soda lignin is entirely free from sulfur, thereby eliminating concerns related to sulfur contamination. It generally shows a higher concentration of phenolic hydroxyl functionalities and a lower molecular weight (76). Additionally, the alkaline treatment often introduces more carboxylic acid groups due to oxidative modifications. However, despite these advantages, soda lignin tends to suffer from significant condensation reactions, which may reduce its reactivity and limit its range of applications. Nonetheless, the sulfur-free nature and relatively high purity of soda lignin make it a valuable candidate for producing bioplastics, adhesives, resins, and various composite materials (77).

#### 3.1.3 Lignosulfonate extraction

Lignosulfonates are produced through the sulfite pulping process, which is performed either under acidic conditions (pH 1–5) or near-neutral conditions (pH 5–7). In acidic environments, lignin first experiences cleavage of *α*-ether bonds, generating quinone methide intermediates. These intermediates subsequently react with bisulfite ions, resulting in the formation of benzyl sulfonic acid groups that greatly enhance lignin’s solubility in water (77, 78). In contrast, under neutral conditions, sulfonation mainly targets the *β*-aryl ether bonds, leading to a different structural modification pathway (79). The lignosulfonate produced through these reactions possesses a combination of hydrophilic groups, including sulfonate, carboxyl, and phenolic hydroxyl groups, alongside hydrophobic aliphatic and aromatic regions, making it highly suitable for use as an anionic surfactant (80).

Following the pulping stage, lignosulfonates are separated from the spent liquor primarily through filtration, often coupled with ultrafiltration techniques to further concentrate and purify the product (81). The molecular weights of lignosulfonates show significant variation depending on the biomass source, with values around 12,000 Da for hardwood-derived lignosulfonates and reaching up to 60,000 Da for those obtained from softwoods (80). Although lignosulfonates are inexpensive and available in large quantities, their broader use is limited by issues such as high sulfur content (ranging from 3 to 8%), structural condensation, and the presence of residual carbohydrates and minerals (82). Despite these drawbacks, their excellent water solubility and favorable surface-active properties have enabled their application as dispersants across multiple industries.

#### 3.1.4 Alkaline and acid pretreatment

**Figure 2:**
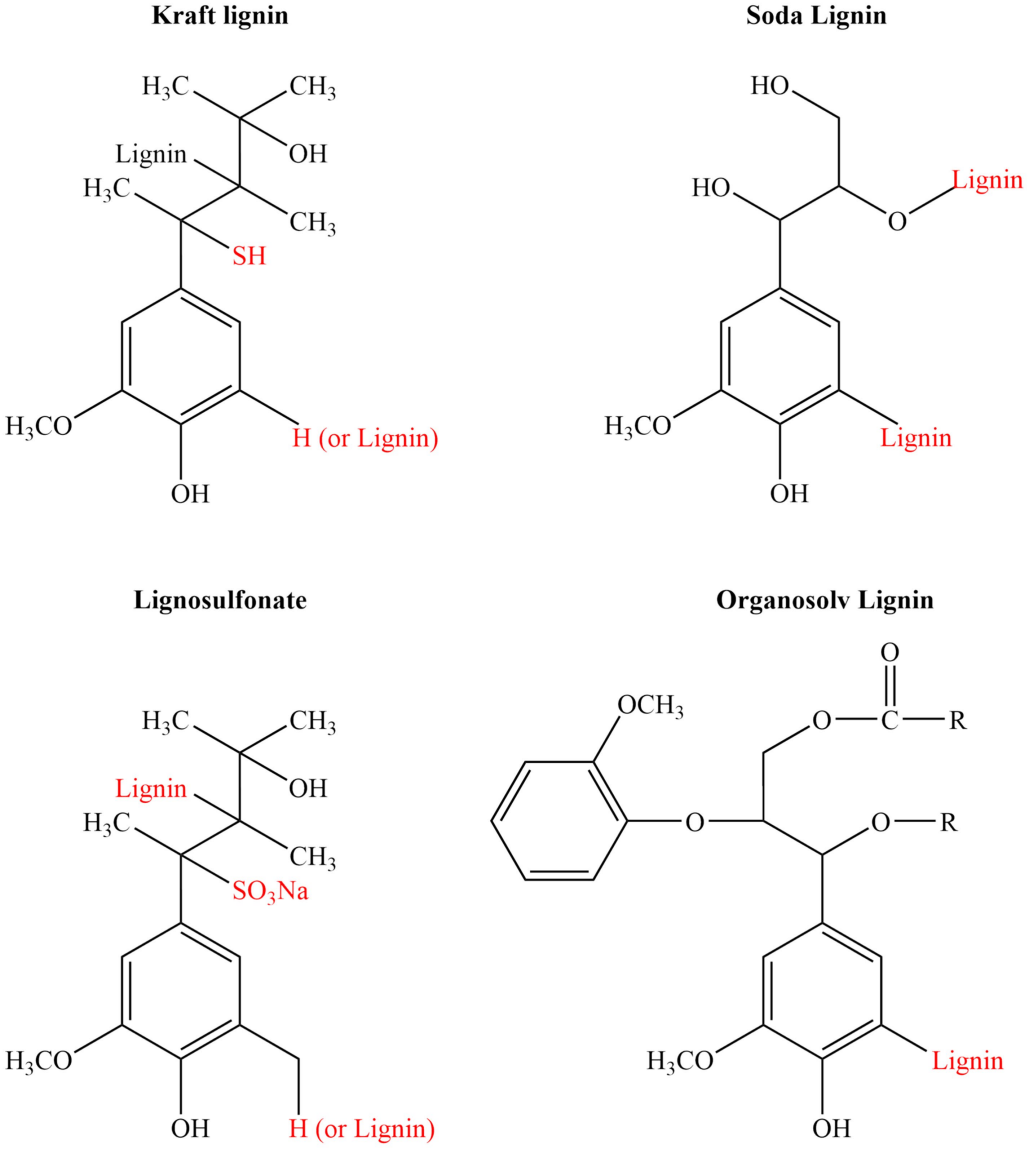
Representative chemical structures of technical lignins derived from major industrial extraction methods: Kraft lignin, Soda lignin, Lignosulfonate, and Organosolv lignin. Each type exhibits characteristic functional groups introduced during the pulping process, such as thiol groups (–SH) in kraft lignin, sulfonate groups (–SO₃Na) in lignosulfonates, or additional hydroxyl groups (–OH) in soda and organosolv lignins, which significantly affect their solubility, reactivity, and applications.

Chemical pretreatment using alkaline or acidic solutions remains one of the most commonly applied strategies for extracting lignin from lignocellulosic biomass. Alkaline pretreatment involves the use of bases such as sodium hydroxide (NaOH), potassium hydroxide (KOH), or ammonium hydroxide (NH₄OH) to break lignin–carbohydrate linkages and solubilize lignin components (83). Conducted under moderate conditions of temperature and pressure, this treatment causes biomass swelling, enhances the accessibility of cellulose fibers, and facilitates the cleavage of ester bonds connecting lignin and hemicellulose structures (83). Among alkaline reagents, NaOH is widely preferred due to its strong alkalinity, broad availability, and relatively low cost (84). One key benefit of alkaline-based extraction is the production of sulfur-free lignin, which is especially suitable for creating bio-based polymers, fuel additives, and other high-value products (85). However, this method is not without drawbacks: alongside lignin removal, there can be unwanted degradation of hemicellulose and cellulose, and significant amounts of alkaline wastewater must be neutralized and managed properly (Figure 2). In contrast, acid pretreatment uses inorganic acids like sulfuric or nitric acid, or organic acids such as acetic or formic acid, to primarily hydrolyze hemicellulose and dissolve acid-soluble fractions of lignin (83). Dilute acid treatments are particularly effective for targeting hemicellulose removal while minimizing cellulose degradation. However, acid pretreatment can lead to the formation of acidifying and toxic compounds, which must be carefully controlled during processing (86).

### 3.2 Organic solvent-based methods

#### 3.2.1 Organosolv lignin extraction

Organosolv lignin is isolated through a relatively eco-friendly method where lignocellulosic materials are delignified using organic solvents such as ethanol, methanol, acetone, ethylene glycol, or various organic acids, typically blended with water (87). In many cases, mineral acids or Lewis acids are introduced as catalysts to enhance the dissolution of lignin and hemicellulose components (88). The extraction process usually operates at temperatures between 160 and 210 °C, with the reaction medium containing more than 60% organic solvent (89). The delignification mechanism primarily involves the cleavage of *β*-O-4 and *α*-ether bonds within the lignin structure, facilitating its release from the biomass matrix (90).

After the pulping step, organosolv lignin is separated by either lowering the pH or cooling the solution, resulting in lignin with relatively low molecular weights, typically around 4,000–10,000 Da, and narrow polydispersity (91). Unlike kraft lignin, organosolv lignin is completely free of sulfur and exhibits high purity, maintaining much of the original structure of native lignin. These features enhance its chemical reactivity and make it suitable for a wide range of applications (71). However, the broader adoption of organosolv processes on an industrial scale faces challenges due to the high cost of solvents, corrosion-related equipment damage, and significant energy demands for solvent recovery. Despite these obstacles, organosolv lignin is highly valued for the development of specialty chemicals, adhesives, and advanced polymer composites (92).

#### 3.2.2 Deep eutectic solvent extraction

Deep eutectic solvents (DESs), formed by combining hydrogen bond donors and acceptors, have emerged as promising green solvents for lignin extraction, offering advantages such as low toxicity, biodegradability, and adjustable properties. In the DES extraction process, the breakdown of lignin’s structure mainly occurs through the cleavage of aryl ether (C–O) and carbon–carbon (C–C) bonds, facilitated by hydrogen bonding interactions and acid-catalyzed solvolysis mechanisms (93). Typically, DESs are prepared through a straightforward method of mixing compounds like choline chloride with organic acids at specific molar ratios, without producing by-products (94). Lignin isolated through DES treatment usually exhibits high purity levels, ranging between 75 and 98%, although minor impurities such as polysaccharide residues and traces of the DES components may remain. The efficiency of lignin extraction and the extent of its depolymerization are highly influenced by factors such as the choice of DES formulation, operational temperature, and the presence of acidic catalysts that assist in protonating *β*-O-4 bonds (95). Importantly, lignin obtained by this method often preserves functional groups crucial for further chemical transformations, making it a valuable raw material for producing antioxidants, sustainable resins, and bio-based composite materials (96).

#### 3.2.3 Ionic liquids extraction

Ionic liquids (ILs) have emerged as promising alternatives for lignin extraction, offering advantages like low volatility, high thermal stability, non-flammability, and strong dissolving power for lignocellulosic components. Their tunable structure—comprising bulky organic cations and variable anions—allows customization for specific biomass treatments (97). Two main approaches are used: direct cellulose dissolution and selective lignin extraction through ionosolv pretreatment. In the ionosolv method, ILs disrupt lignin–carbohydrate bonds, enabling lignin removal while largely preserving cellulose integrity (98). Recent advances in designing functionalized ILs have further improved lignin solubility and selectivity. Protic ILs, in particular, enhance *β*-O-4 bond cleavage, crucial for generating valuable lignin-based products (99). Despite their potential, IL technologies face hurdles such as high production costs, toxicity concerns, and complex solvent recovery. However, ongoing efforts to develop biodegradable and affordable ILs are bringing this technology closer to large-scale lignin valorization (100).

### 3.3 Physicochemical and assisted extraction techniques

The development of sustainable lignin isolation strategies has gained increasing interest as industries seek environmentally friendly methods for biomass valorization. While several techniques are often described as green, it is important to clarify that physical-assisted methods such as microwave-assisted extraction (MAE) and plasma-assisted treatment are not inherently green unless paired with appropriate chemical systems. These technologies serve primarily as process intensification tools that can improve extraction efficiency when used in conjunction with alkaline, acidic, or deep eutectic solvents. The actual environmental footprint of these methods depends heavily on the nature of solvents and reagents employed.

#### 3.3.1 Supercritical fluid extraction

Supercritical fluid extraction (SFE) has emerged as a promising physicochemical approach for lignin isolation, offering reduced use of organic solvents, high selectivity, and potential for solvent recovery. In this method, fluids above their critical temperature and pressure exhibit enhanced diffusivity and tunable solvation capabilities, allowing deep penetration into lignocellulosic matrices and facilitating lignin dissolution (101). Supercritical carbon dioxide (SC-CO₂) is the most commonly used fluid due to its mild critical parameters (31.1 °C, 73.8 bar), low toxicity, and ease of recycling (84). However, SC-CO₂ alone exhibits limited solubility for lignin, and is often combined with co-solvents such as ethanol or methanol to enhance lignin extraction efficiency and disrupt hydrogen bonding networks. Studies report lignin yields exceeding 70% in SC-CO₂–ethanol systems, particularly when process parameters are optimized (102, 103). Additionally, supercritical water has been explored as an alternative medium for lignin depolymerization. Although SFE is associated with high initial equipment costs and energy demands to maintain supercritical conditions, its ability to produce sulfur-free lignin of high purity makes it an attractive candidate for scalable biorefinery applications (104).

#### 3.3.2 Microwave-assisted extraction

Microwave-assisted extraction (MAE) is widely investigated for accelerating lignin release by applying high-frequency electromagnetic radiation to biomass. Microwave energy penetrates biomass particles and induces rapid dielectric heating via dipolar rotation and ionic conduction, which causes localized rupture of the cell wall and enhances mass transfer (105). However, MAE alone is not sufficient for effective lignin extraction and must be used in combination with chemical agents, typically alkaline (e.g., NaOH) or acidic solutions, that actively cleave ester and ether bonds within the lignocellulosic structure (106). The role of microwave energy in these hybrid systems is to enhance molecular motion, accelerate reaction kinetics, and improve solvent diffusion. More recently, green solvents such as deep eutectic solvents (DESs) have been explored in MAE systems to reduce environmental burden while maintaining high efficiency (107, 108). Despite limitations such as non-uniform heating and specialized reactor requirements, MAE remains a valuable process intensification strategy when integrated with sustainable chemical formulations. Its advantages include shorter processing times, reduced solvent consumption, and potential retention of lignin’s functional moieties (109).

#### 3.3.3 Plasma-assisted extraction

Plasma-assisted pretreatment is a novel approach that utilizes ionized gases to alter biomass structure through chemical and physical interactions. Plasma, comprising electrons, ions, UV photons, and reactive oxygen or nitrogen species, can induce bond scission and surface modification of lignocellulosic substrates (110). Non-thermal plasma (NTP), in particular, has gained attention due to its operation at near-ambient temperatures, minimizing thermal degradation. However, like MAE, plasma treatment alone does not suffice for efficient lignin solubilization, and is generally combined with alkaline or oxidizing agents to facilitate effective delignification. For example, applying NTP to alkali-impregnated biomass significantly improves lignin removal by enhancing hydroxyl radical formation and disrupting recalcitrant bonds (111, 112). In such integrated systems, plasma accelerates depolymerization reactions, reduces inhibitor formation, and improves enzyme accessibility in downstream processes. While current research has demonstrated high delignification rates and improved accessibility for saccharification, key challenges remain in energy efficiency, reactor design, and scale-up feasibility (113–115). However, challenges like optimizing energy efficiency, reactor design, and scaling up remain. Although still under development, plasma-assisted delignification holds strong potential as a green and efficient alternative for future lignin extraction technologies (110). As such, plasma-assisted lignin pretreatment is better considered a complementary technique rather than a standalone green extraction method.

### 3.4 Biological/enzymatic extraction

#### 3.4.1 Enzymatic hydrolysis lignin

Enzymatic hydrolysis lignin, often referred to as cellulolytic enzyme lignin (CEL), is obtained as a solid residue following the enzymatic breakdown of lignocellulosic biomass. In this process, cellulase, and hemicellulase enzymes are employed to selectively hydrolyze polysaccharides (cellulose and hemicellulose) under mild, aqueous, and environmentally benign conditions, leaving lignin as an unreacted component. These conditions largely preserve lignin’s native structure and avoid the introduction of sulfur or other chemical contaminants typically associated with pulping or harsh chemical pretreatments (116, 117). However, the crude residue resulting from enzymatic hydrolysis still contains entrapped carbohydrates, proteins, and ash, which can interfere with downstream applications or structural characterization. Therefore, to obtain purified CEL, the lignin-rich fraction is typically subjected to solvent extraction using dioxane–water mixtures (commonly 96:4 v/v). This step efficiently dissolves lignin while leaving behind polysaccharides and other insoluble impurities. The resulting CEL exhibits relatively high lignin content (typically 65–80%) and retains important native features such as phenolic hydroxyls, *β*–O–4 linkages, and aromatic ring structures, making it valuable for detailed structural analysis and functionalization (73, 118, 119). Although CEL is poorly soluble in water and many conventional organic solvents, its chemical integrity and absence of harsh modifications make it a promising candidate for diverse applications. These include its use as a dispersant, binder, adsorbent, antioxidant additive, or renewable precursor in polymer and composite synthesis. Further chemical modification or nanoparticle formulation may be required to overcome solubility challenges in specific applications.

### 3.5 Specialized lignin type extraction

#### 3.5.1 Catechyl lignin

Catechyl lignin (C-lignin) is a recently identified type of lignin, predominantly located in the seed coats of certain plants such as castor, vanilla orchid, and cactus (120). Unlike conventional lignins, which are composed of a mixture of guaiacyl, syringyl, and p-hydroxyphenyl units, C-lignin is exclusively formed from caffeyl alcohol monomers connected through benzodioxane bonds. This distinct linear structure results in a highly uniform and less branched polymer compared to the complex, heterogeneous architecture of typical lignins (121). The presence of stable benzodioxane linkages grants C-lignin exceptional resistance to chemical degradation, especially under acidic environments. This structural stability allows C-lignin to undergo clean and efficient depolymerization into catechol-based products using relatively straightforward catalytic processes. Extraction of C-lignin has been effectively achieved using deep eutectic solvents, particularly from biomass sources like castor seed coats, producing lignin fractions with low molecular weights (ranging from 1,800 to 3,500 Da) and narrow polydispersity. Due to its simple depolymerization pathway and the potential to yield valuable catecholic compounds, C-lignin is regarded as an attractive option for future sustainable lignin valorization efforts (122, 123).

Therefore, the extraction of lignin from lignocellulosic biomass encompasses a broad range of traditional and emerging technologies, each offering distinct advantages and challenges. Conventional methods such as kraft, sulfite, soda, and organosolv processes have been widely implemented, yielding large volumes of technical lignins with varying structural properties. Innovations including deep eutectic solvents, ionic liquids, microwave-assisted extraction, and plasma-assisted techniques have been developed to enhance lignin purity, reduce environmental impact, and preserve native lignin structures. Despite the progress achieved, challenges such as scalability, cost-effectiveness, solvent recovery, and process optimization remain critical barriers to the widespread industrial application of newer methods. Nevertheless, advances in green solvent technologies, supercritical fluids, and plasma-based systems offer promising avenues for sustainable lignin extraction, enabling the production of high-value lignin suitable for a wide range of industrial and biotechnological applications.

### 3.6 Recyclability and long-term performance of LNPs

While much of the current research has focused on the functional performance of LNPs in biopolymer films, there is a notable lack of experimental data on how these materials behave during or after recycling processes. In particular, it is unclear how thermal, mechanical, or chemical recycling methods affect the structural integrity, antioxidant activity, or barrier properties of LNPs once incorporated into food packaging matrices. As LNPs are biodegradable and often sensitive to heat and oxidation, their performance may degrade with repeated processing. However, no comprehensive studies have quantified these changes under real-world recycling conditions. This represents a critical knowledge gap in validating the role of LNP-based packaging within circular economy frameworks. Future investigations should assess the retention of nanoparticle functionality after recycling, evaluate nanoparticle migration behavior in reprocessed films, and explore strategies to enhance LNPs recyclability in bio-based composites.

### 3.7 Influence of extraction methods on LNPs properties

The extraction method applied to isolate lignin from biomass significantly influences the physicochemical characteristics of the resulting lignin, which in turn governs its behavior during nanoparticle formation and the properties of the produced LNPs. Variations in extraction severity, chemical environment, and process conditions lead to lignin fractions with different molecular weights, functional group profiles, condensation degrees, and residual carbohydrate contents. These compositional and structural differences have critical implications for nanoparticle morphology, stability, and potential end-use functionality (124). For example, lignins obtained through relatively harsh treatments such as kraft pulping tend to have higher molecular weights, a greater degree of condensation, and more aliphatic and phenolic hydroxyl groups exposed due to ether bond cleavage (125). Despite its structural heterogeneity and lower solubility, kraft lignin has demonstrated exceptional potential for producing small-sized, monodisperse LNPs with high colloidal stability (126). The presence of abundant carboxylic acid groups and possible co-extracted hydrophobic compounds, such as resinous by-products from pulping, appear to facilitate the formation of nanoparticles with low diameters and strong negative zeta potentials, without the need for added surfactants or stabilizing agents (127).

On the other hand, lignins extracted via milder organosolv or acid-catalyzed methods typically retain a larger proportion of native ether linkages, especially *β*-O-4 bonds, and show lower levels of structural degradation. These lignins usually possess a narrower molecular weight distribution and higher chemical purity, which make them well-suited for applications where biocompatibility and reactivity are prioritized (128, 129). However, the resulting nanoparticles from these lignins often exhibit slightly larger sizes (ranging from approximately 60 to 100 nm), broader size distributions, and somewhat reduced colloidal stability compared to those derived from kraft lignin (130–132). Adjusting the pH during nanoprecipitation has been shown to be a simple yet highly effective strategy for tuning LNPs characteristics. Increasing the pH of the lignin solution prior to solvent shifting leads to deprotonation of carboxylic and phenolic groups, enhancing electrostatic repulsion and promoting the formation of smaller and more stable nanoparticles (133). The use of neutral to slightly basic conditions during nanoparticle precipitation has consistently resulted in reduced particle size and improved dispersion stability across multiple lignin types (127, 132).

Overall, while all major classes of technical lignin can be converted into nanoscale dispersions using appropriate techniques, the properties of the resulting LNPs are intricately tied to the origin and extraction pathway of the precursor lignin. Lignins derived from kraft processes may be more suitable for applications that require small, thermally stable nanoparticles with narrow size distributions, whereas lignins isolated through organosolv or acidolysis routes may offer advantages in terms of functional group availability, purity, and biocompatibility. The interplay between extraction conditions, molecular structure, and nanoparticle formation underscores the importance of tailoring lignin selection and processing strategies to match the performance requirements of specific material applications.

## 4 Lignin nanoparticles synthesis techniques for food packaging applications

**Figure 3:**
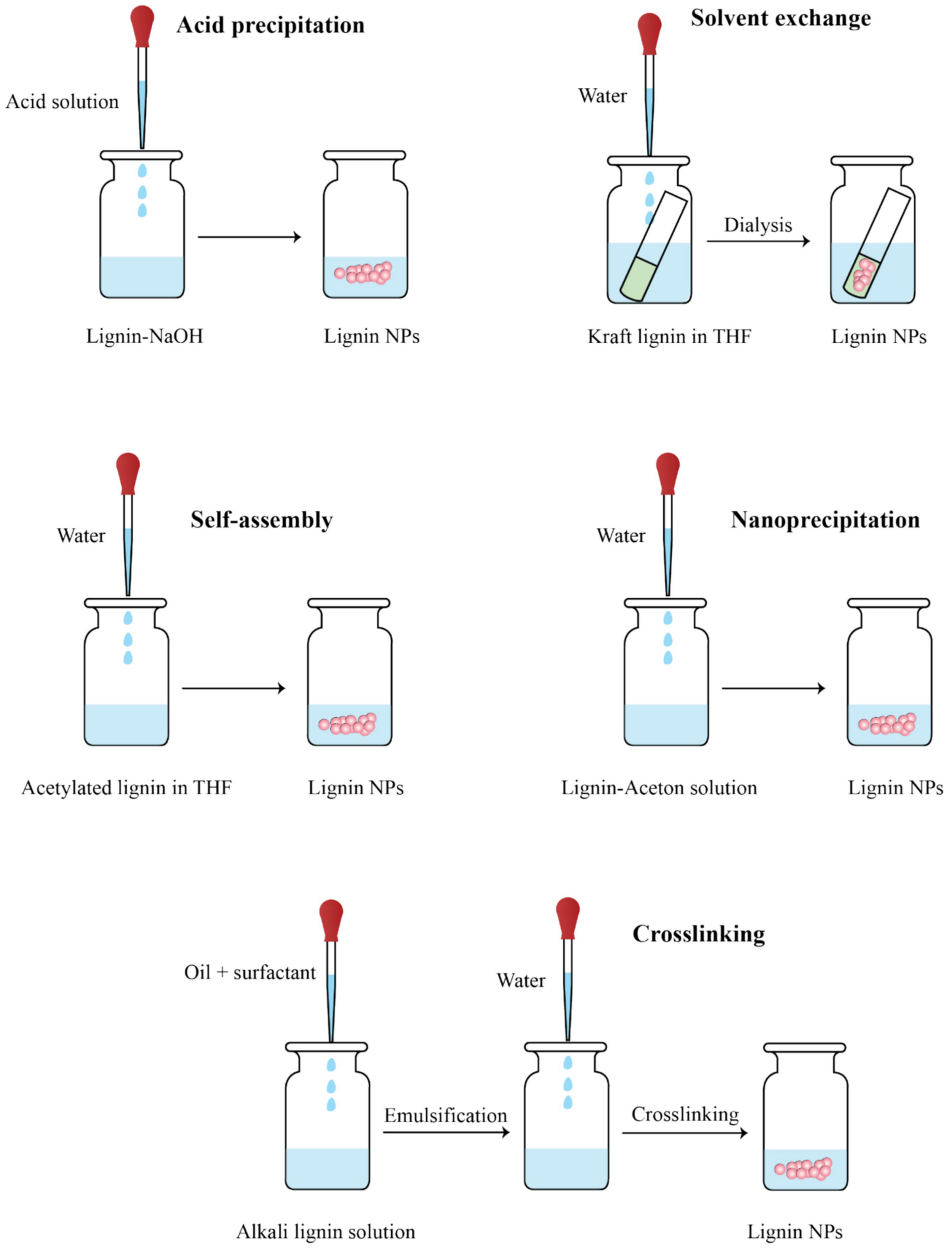
Schematic representation of six major methods for synthesizing lignin nanoparticles (LNPs), including acid precipitation, solvent exchange, self-assembly, nanoprecipitation, and interfacial crosslinking.

The synthesis of LNPs has gained increasing attention due to its critical influence on particle morphology, size, surface chemistry, and colloidal stability, all of which directly affect their performance in food packaging applications (Figure 3). A wide range of fabrication techniques has been developed, including mechanical, physicochemical, chemical, and biological approaches. Among these, methods such as antisolvent precipitation, homogenization, and electrospray have already been successfully applied to produce LNPs that, when incorporated into food packaging films, have demonstrably improved their mechanical strength, barrier properties, UV shielding, antioxidant activity, and antimicrobial function, as confirmed in numerous studies detailed below and in section 5. In contrast, several other strategies such as enzymatic modification, polymer grafting, or aerosol flow reactors are still in early development for lignin systems and have not yet been applied in packaging contexts. Table 2 summarizes the underlying principles, advantages, and limitations of these synthesis methods. To bridge synthesis conditions with material functionality, this section also highlights selected examples where specific LNPs fabrication methods have resulted in measurable improvements in packaging performance. These interconnections are further elaborated in Table 3, which compiles published studies on the application of LNPs in packaging systems.

**Table 2:** Overview of lignin nanoparticle (LNP) synthesis techniques.

Synthesis technique	Process principle	Key features	Advantages	Limitations
Antisolvent precipitation	Inducing nanoparticle formation by decreasing lignin solubility with a poor solvent	Fast precipitation; uniform spheres	Simple, scalable, no harsh reagents	Risk of aggregation; particle size variation
Solvent exchange (dialysis)	Gradual solvent substitution causing nanoparticle self-assembly	High purity, stable dispersions	Green method, solvent removal avoids aggregation	Time-consuming, expensive membranes
Ultrasonication	Acoustic cavitation breaks macroaggregates into nanoparticles	Irregular to spherical morphology	Simple, chemical-free, rapid	Broad size distribution; oxidation risk
Self-assembly	Spontaneous aggregation of lignin via hydrophobic and hydrogen bonding	Micellar or hollow structures	Controlled morphology, tunable size	Needs organic solvents; limited pH stability
Polymerization-grafting	Chemical modification followed by self-organization into nanoparticles	Core-shell or porous structures	Functionalized surfaces, targeted applications	Requires toxic initiators or solvents
Ionic crosslinking	Formation of crosslinked nanoparticles at oil/water interfaces	Hollow nanocapsules	Encapsulation capability, stable structures	Toxic crosslinkers sometimes needed
Supercritical CO₂ precipitation	Rapid lignin supersaturation by solvent removal in SC-CO₂	Small, uniform particles	Solvent-free final product, green process	High pressure equipment needed, expensive
Aerosol flow reactor	Atomization and thermal drying of lignin droplets into particles	Controlled size, dry powder	Continuous production, scalable	Requires reactor optimization
Ball milling	Mechanical grinding reduces lignin to nanoscale	Irregular shapes; broad size range	Solvent-free, low cost	Potential contamination; less uniform size
Spray freezing into liquid nitrogen	Rapid freezing of lignin solution droplets	Nanoporous or irregular structures	Simple, no chemical additives	Poor size control; low homogeneity

**Table 3:** An overview of studies on application of lignin nanoparticles in food packaging.

Polymer matrix	Method	Properties improved	Application	References
Chitosan	Antisolvent precipitation	Tensile strength, oxygen/water barrier, UV blocking, antibacterial	Antibacterial food packaging	(157)
PVA	Antisolvent precipitation (DES)	Thermal stability, mechanical strength, hydrophobicity, UV shielding, antioxidant, antibacterial	Active food packaging	(156)
PVA	Antisolvent precipitation + loading with potassium sorbate	Tensile strength, oxygen/water barrier, antibacterial, UV blocking	Active food packaging (strawberry preservation)	(195)
PE (polyethylene)	Layer-by-layer assembly	Oxygen and water vapor barrier, light barrier	Coatings for food packaging	(179)
Paper (cellulose)	Homogenization	Water/oil repellency, water vapor barrier, tensile strength	High-barrier packaging	(137)
PVA	Solvent casting	Thermal stability, antioxidant, tensile strength, water barrier	Active food packaging	(196)
Polysaccharides (e.g., pectin, starch)	Coating (Polydopamine modification)	Mechanical strength, UV-blocking, antioxidant, antimicrobial, flexibility	Active coatings for fruits	(189)
Casein	Eco-friendly acid hydrotrope extraction	Mechanical strength, UV blocking, antioxidant, antibacterial, recyclability	Active biodegradable packaging	(191)
PVA/Chitosan	Solvent casting	Tensile strength, UV blocking, antioxidant, antibacterial	Antibacterial food packaging	(197)
PLA	Electrospray	UV-blocking, antioxidant, mechanical enhancement	Food packaging	(164)
PLA	Melt extrusion	Transparency, UV blocking, mechanical strength, antibacterial	Antibacterial food packaging	(202)
Chitosan	Antisolvent precipitation + coating	Antibacterial, UV shielding, water resistance	Eco-friendly antimicrobial packaging	(177)
Chitosan	Antisolvent precipitation (DES)	Tensile strength, UV shielding, antioxidant, shelf-life extension	Refrigerated fish preservation	(158)
PBAT	Melt compounding with LNPs-TiO₂	Mechanical strength, barrier, antimicrobial	Food packaging	(207)
PBS	Solvent casting	Thermal stability, mechanical strength, barrier, antifungal	Active packaging (bread)	(204)
PBAT	Hydrothermal hybridization with ZnO	UV blocking, antioxidant, antibacterial, mechanical strength	Active food packaging	(206)
PLA	Extrusion	UV blocking, antioxidant, compostability	Compostable food packaging	(203)
PHBV	Electrospinning + film formation	UV blocking, antioxidant, antibacterial, oxygen barrier	Sustainable food packaging	(208)
Starch	Antisolvent precipitation	Mechanical properties, UV shielding, antioxidant	Advanced food packaging	(182)
PVA	Solvent casting	Thermal stability, toughness, hydrophobicity, UV shielding	Biodegradable food packaging	(199)
PLA	Organocatalyzed lactide polymerization	UV blocking, antioxidant, dispersion stability	Food packaging	(170)

### 4.1 Top-down mechanical methods

#### 4.1.1 Homogenization

Homogenization is a mechanical method for synthesizing LNPs by applying intense shear forces to disrupt lignin aggregates without chemical modification (134). In this process, lignin is dispersed in solvents like water or ethanol and subjected to high-speed shearing, where cavitation effects fragment the particles. Solvent choice critically influences cavitation intensity and thus nanoparticle size and morphology. Ethanol–water systems, particularly with pre-treatment or acidic catalysts (as in HOS-SE), enhance size reduction and particle reshaping (135). Compared to ultrasonication, homogenization yields smaller, more uniformly sized nanoparticles while preserving lignin’s chemical structure (136). Prolonged treatment improves colloidal stability by minimizing agglomeration (135). The resulting LNPs also exhibit high thermal stability, and the method’s simplicity, chemical-free nature, and scalability make it highly suitable for industrial applications, provided that operational conditions are carefully optimized. A representative application of LNPs prepared via homogenization is their incorporation into cellulose-based paper coatings, where they enhanced water and oil repellency, improved tensile strength by 48%, and reduced water vapor permeability by over sixfold, demonstrating their effectiveness as multifunctional barrier layers (137).

#### 4.1.2 Ultrasonication

Ultrasonication is an efficient, green method for producing LNPs by using acoustic cavitation to induce particle size reduction, bond cleavage, and oxidation without major chemical alteration. Its effectiveness depends on sonication time, power, solvent type, pressure, and temperature (138). Higher ultrasonic intensities and optimized conditions yield smaller, more uniform LNPs, whereas insufficient intensity leads to broader size distributions and irregular morphologies (139). Ultrasonication also promotes functional group exposure, enabling complex formation with other molecules, such as gelatin and chitosan (140, 141). Excessive sonication can cause lignin degradation or, alternatively, radical-induced crosslinking, depending on conditions (142). Advanced techniques like ultrasonic-assisted solvent shifting enhance particle uniformity (143). Despite challenges like non-uniform particle sizes under suboptimal conditions, ultrasonication remains a versatile, scalable, and environmentally friendly approach for nanostructured lignin production (144, 145).

#### 4.1.3 Ball milling

Ball milling is a simple, solvent-free mechanical technique used to reduce lignin particle size by applying impact and shear forces through rotating balls within a cylindrical shell (146). Different types of mills, including tumbler, vibratory, and planetary mills, enable the efficient breakdown of lignin depending on milling parameters such as ball size, rotational speed, and temperature (147).

Modified ball milling methods, like low-temperature milling, have produced LNPs smaller than 10 nm (148). Despite its advantages of low cost, simplicity, and scalability, this method can result in size variation, broad particle distributions, and contamination risks (149). Nonetheless, ball milling remains a practical approach for large-scale lignin nanoparticle production.

### 4.2 Solvent-based precipitation methods

#### 4.2.1 Antisolvent precipitation method

Antisolvent precipitation is a simple and effective approach for synthesizing LNPs by reducing lignin solubility through the addition of water or acid into lignin solutions prepared in organic solvents like THF, ethanol, acetone, or DMSO. This induces rapid aggregation, yielding spherical nanoparticles with smooth surfaces and uniform sizes (150, 151). Lignin properties, including molar mass, hydroxyl content, and solubility, significantly affect nanoparticle formation. Process parameters such as lignin concentration, solvent/antisolvent ratio, and pH control the particle size and stability (152). For example, adjusting pH in sodium p-toluenesulfonate solutions can reduce particle size by promoting dissociation of lignin functional groups (148). Acid precipitation using HCl or HNO₃ also forms stable LNPs by protonating lignin, improving stability and imparting antibacterial properties (153, 154). While environmentally friendly and cost-effective, this method may cause aggregation during solvent removal if not carefully controlled (155). Overall, antisolvent precipitation produces functional, biodegradable LNPs suitable for food packaging, cosmetics, and drug delivery. Antisolvent precipitation has been widely used to fabricate well-dispersed LNPs for bioactive films. For instance, chitosan and PVA films incorporating LNPs synthesized via this method exhibited marked improvements in mechanical strength (up to 55%), UV-blocking, and antibacterial activity (e.g., >99% inhibition against *E. coli* and *S. aureus*) (156, 157). When applied to refrigerated fish preservation, these films prolonged shelf life by up to 4 days compared to controls (158).

#### 4.2.2 Solvent exchange method

The solvent exchange method is a simple and chemical-free approach for LNPs synthesis, where lignin is first dissolved in an organic solvent like THF or DMSO, then gradually dialyzed against water to induce nanoparticle formation (127, 159). Lievonen et al. produced stable LNPs by dissolving kraft lignin in THF followed by water dialysis, with particle size tunable by adjusting lignin concentration [8]. Lintinen et al. (159) prepared metal–organic nanoparticles by introducing iron-isopropoxide into lignin/THF solutions before dialysis [6]. Figueiredo et al. (71) extended this approach by forming Fe₃O₄–lignin hybrid nanoparticles. Similarly, Zikeli et al. (160) generated LNPs from wood waste lignin dissolved in DMSO and dialyzed against water. While the method offers high nanoparticle stability and simplicity, the need for dialysis membranes and solvent handling can increase production costs.

### 4.3 Spray and aerosol techniques

#### 4.3.1 Electrospinning method

Electrospinning uses a high-voltage electric field to create a fine jet of lignin solution, forming continuous nanofibers as the solvent evaporates. Typically, the lignin solution is loaded in a syringe connected to the positive electrode, while the collector is attached to the negative electrode. Upon applying voltage, the solution forms a thin stream that solidifies into fibers (161). Ruiz-Rosas et al. (162) produced lignin submicron fibers by electrospinning Alcell lignin/ethanol mixtures, achieving good oxidation resistance and microporous carbon fibers after stabilization and carbonization. Dallmeyer et al. (163) evaluated various lignins (kraft, organosolv, sulfonated, pyrolytic) for electrospinning and found that fiber formation was improved by adding poly(ethylene oxide) (PEO), enabling uniform fibers. Overall, electrospinning allows the fabrication of defect-free, thermally stable lignin fibers, though fiber morphology is influenced by lignin type, solution properties, and solvent choice. Electrospray-based LNPs fabrication enables uniform nanoparticle deposition and has shown strong application potential in PLA films. These films displayed excellent UV-blocking ability (transmittance below 1.1% at 280 nm), antioxidant enhancement (10-fold increase in DPPH scavenging), and improved mechanical integrity, validating the role of this synthesis method in producing optically active and oxidation-resistant packaging layers (164).

#### 4.3.2 Spray freezing

Spray freezing involves dissolving lignin in a solvent such as DMSO and rapidly spraying the solution onto a surface cooled by liquid nitrogen. Upon contact, the lignin droplets instantly freeze, forming solid particles without extensive solvent-lignin interaction. This method offers simple, continuous nanoparticle production and avoids chemical modifications, but typically results in particles with heterogeneous size and morphology (165).

#### 4.3.3 Aerosol flow reactor synthesis

The aerosol flow reactor technique offers a continuous, one-step method for LNPs production with uniform size distribution. In this process, lignin is dissolved in a solvent like water or DMF, atomized into fine droplets, and carried by a nitrogen gas stream through a heated laminar flow reactor, where solvent evaporation yields solid nanoparticles. Ago et al. (166) demonstrated that lignin concentration influences particle size and dispersion, with higher concentrations producing larger but more narrowly distributed LNPs. The resulting particles showed excellent mechanical integrity and stable redispersibility in oil/water systems, efficiently stabilizing Pickering emulsions. This method is valued for its simplicity, scalability, high product yield, and minimal liquid waste generation (166).

### 4.4 Polymerization and crosslinking strategies

#### 4.4.1 Polymerization method

Polymerization techniques for LNPs often involve grafting polymer chains onto lignin to improve functionality. Barakat et al. (167) synthesized nanoparticles from arabinoxylan–dehydrogenation polymers through polymerization of coniferyl and sinapyl alcohols in the presence of heteroxylans. Qian et al. (168) grafted 2-(diethylamino)ethyl methacrylate onto alkali lignin via ATRP, enabling switchable Pickering emulsions. Other approaches, such as miniemulsion polymerization, produced lignin nanocarriers with various morphologies, where porous nanoparticles exhibited faster release profiles. Further, solvent-free radical polymerization has been used to create lignin–PMMA composites, improving mechanical, thermal, and UV-resistant properties. Grafting PMMA onto lignin by ATRP or radical polymerization enhanced miscibility with other polymers and promoted biomedical applications (169). Although polymerization improves lignin compatibility and functionality, it often involves costly and hazardous reagents. Polymer-grafted LNPs obtained via organocatalyzed ring-opening polymerization of lactide displayed enhanced compatibility in PLA matrices. These hybrid materials demonstrated superior nanoparticle dispersion, increased UV-shielding (up to 85% reduction in UV-A transmission), and sustained antioxidant release profiles over time, enabling their use in intelligent or active packaging systems (170).

#### 4.4.2 Emulsion and crosslinking methods

Emulsion and crosslinking techniques have been widely applied to synthesize lignin-based nanocapsules and microspheres. Typically, lignin is first emulsified in an oil/water system and stabilized using surfactants, followed by crosslinking at the droplet interfaces to form stable nanostructures. Yiamsawas et al. (171) developed hollow lignin nanocapsules by crosslinking sodium lignosulfonate and alkali lignin using toluene diisocyanate (TDI) at the miniemulsion interface, resulting in stable capsules suitable for aqueous or organic dispersions. Similarly, application of ultrasound-assisted crosslinking in oil/water emulsions formed kraft lignin microcapsules with biocompatibility properties (171). Chemical crosslinkers like epichlorohydrin have been used to form porous lignosulfonate spheres with enhanced porosity and mechanical properties. Lignin supracolloids also synthesized through microemulsion formation followed by crosslinking. Similarly, pH-responsive lignin nanocapsules fabricated via interfacial miniemulsion polymerization (172–174). Overall, these methods enable the formation of structurally stable, functional lignin nanoparticles with potential applications in controlled release systems, packaging, and biomedicine.

### 4.5 Biological and enzymatic methods

#### 4.5.1 Biological method

The biological method for LNPs synthesis utilizes enzymes or microorganisms to break down lignocellulosic structures and release lignin, offering a green and low-cost approach. Accordingly, cuboidal LNPs has been produced by enzymatically hydrolyzing Indian ridge gourd lignocellulose. Similarly, combination of enzymatic hydrolysis with a solvent exchange process led to prepare stable LNPs from various biomasses. Although the biological route avoids hazardous chemicals and is environmentally sustainable, it typically results in low nanoparticle yield, irregular shapes, and larger particle sizes. Nevertheless, this method remains attractive for sustainable nanomaterial production where eco-friendliness is prioritized (174, 175).

Thus, various physical, chemical, and biological strategies have been employed for LNPs synthesis, each offering specific advantages and limitations. Mechanical approaches such as ultrasonication, homogenization, and ball milling provide simplicity but may lead to broad particle size distributions. Techniques based on self-assembly, antisolvent precipitation, solvent exchange, and aerosol flow reactors enable better control over particle morphology and dispersibility, although process optimization remains crucial. Crosslinking and polymerization methods enhance the structural integrity and functional versatility of LNPs, whereas biological and dialysis routes offer greener alternatives with minimal chemical input. Ice segregation-induced self-assembly and spray freezing present emerging methods that capitalize on environmentally friendly conditions for nanoparticle fabrication. Overall, the selection of the appropriate synthesis method depends on the desired particle size, stability, functionalization potential, and application field, particularly in sustainable food packaging.

In summary, this section illustrates that the choice of LNP synthesis technique has a direct impact on packaging film performance. Methods like antisolvent precipitation, electrospray, and polymer grafting yield nanoparticles with defined morphologies, surface functionalities, and dispersion characteristics that significantly enhance film strength, antioxidant capacity, UV resistance, and antimicrobial behavior. These correlations between synthesis and functionality are critical for designing high-performance food packaging systems, as further demonstrated in section 5.

## 5 Applications of lignin nanoparticles in food packaging

The integration of LNPs into biodegradable polymer matrices has emerged as a promising strategy for enhancing the functional performance of food packaging materials. Owing to their intrinsic antioxidant, UV-blocking, and antimicrobial properties, as well as their ability to form strong interfacial interactions, LNPs significantly improve the mechanical strength, barrier properties, and functional bioactivity of biodegradable films. Various biodegradable polymers, including polysaccharides, proteins, and polyesters, have been reinforced with LNPs to develop active and intelligent food packaging systems that meet both sustainability objectives and regulatory standards. Among these matrices, chitosan, polyvinyl alcohol (PVA), polylactic acid (PLA), and polybutylene succinate (PBS) have received particular attention due to their film-forming ability and environmental compatibility (Table 3). This section categorizes and discusses recent advances in LNPs-enhanced biodegradable packaging materials, organized by polymer type, beginning with chitosan-based systems.

## 6 Applications of lignin nanoparticles in sustainable food packaging

### 6.1 Chitosan-based systems (CH matrix)

Chitosan (CH), a naturally derived polysaccharide obtained from chitin deacetylation, is one of the most promising candidates for replacing petroleum-derived plastics in sustainable food packaging. Its film-forming ability, biodegradability, biocompatibility, and intrinsic antimicrobial activity make it highly attractive. Nonetheless, native chitosan films suffer from low mechanical strength, limited oxygen and UV barrier properties, and high water vapor permeability, which restrict broader industrial adoption.

To overcome these limitations, numerous studies have explored the incorporation of LNPs into chitosan matrices. LNPs offer multiple functionalities, including UV shielding, antioxidative activity, moisture resistance, and mechanical reinforcement. This combination creates synergistic nanocomposites with markedly enhanced packaging performance.

**Figure 4:**
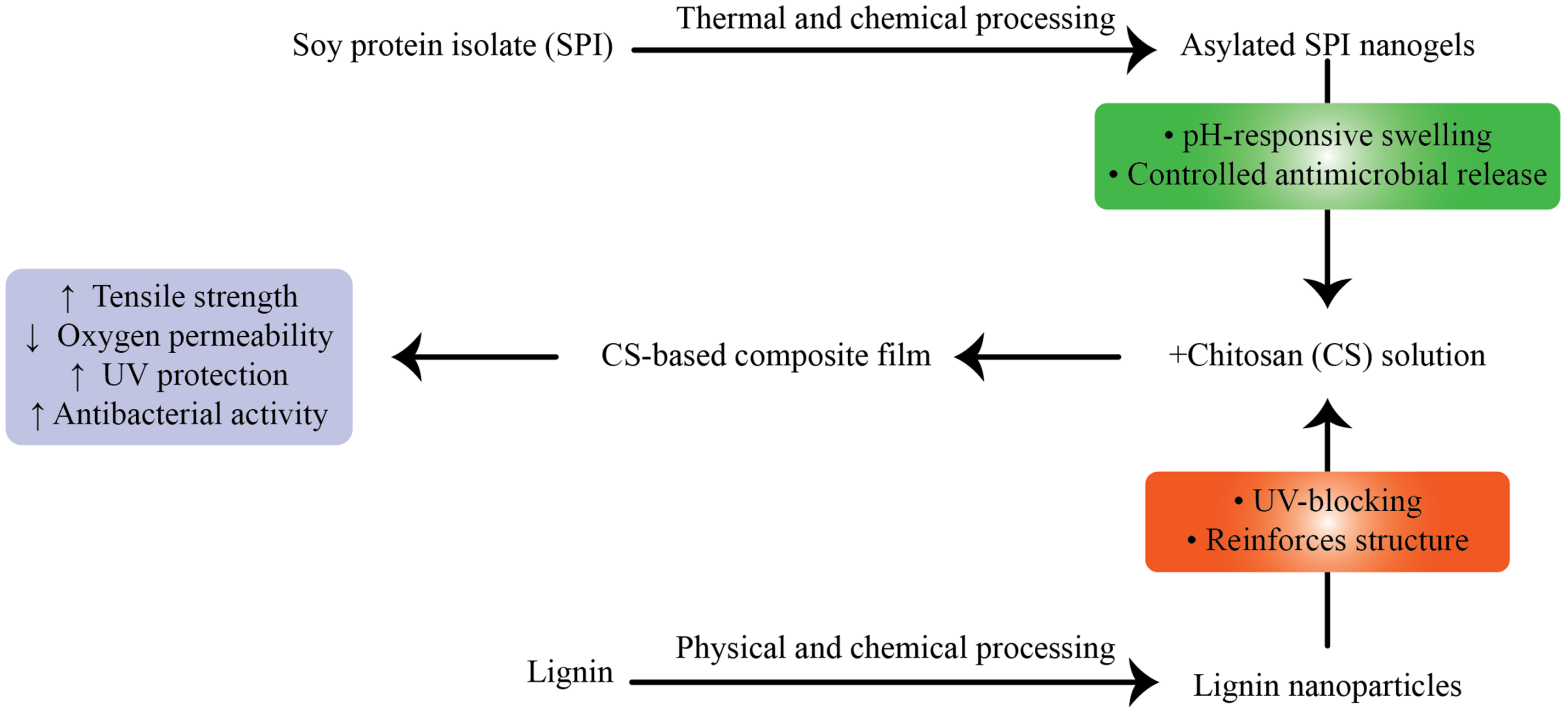
Preparation and characterization of chitosan (CS) composite films incorporating lignin nanoparticles (LNPs) and acylated soy protein isolate nanogels (ASPNG). The co-incorporation improved mechanical strength, barrier properties, UV-blocking, and antibacterial activity. FT-IR spectra and UV-transmittance curves confirmed functional enhancements, while SEM imaging showed uniform dispersion of LNPs and ASPNG within the CS matrix.

Zou et al. (157) developed CH films containing LNPs and acylated soy protein isolate nanogels (ASPNG), which significantly improved film tensile strength (from 37.29 to 54.29 MPa), reduced oxygen permeability, and imparted strong antibacterial properties (Figure 4). The co-incorporation strategy demonstrated a synergistic effect, with ASPNG providing controlled release and LNPs reinforcing the film structure while also improving UV protection (157). In a separate study, Zhang et al. (176) prepared chitosan nanoparticle-based films (NCH) reinforced with alkali LNPs. The resulting NCH–LNPs composites showed increased crystallinity and thermal stability, along with a 1.5–3.4-fold increase in antioxidant capacity relative to controls. Applied to grape and cheese preservation, these films reduced lipid peroxidation and extended shelf life, underscoring their translational utility (176).

A similar enhancement was observed by Winotapun et al. (177), who applied CH–LNPs coatings onto bagasse paper using fractionated kraft lignin. The coatings showed nearly complete bacterial inhibition (>2 log CFU/cm^2^ reduction against *S. aureus* and *E. coli*), as well as enhanced hydrophobicity. These eco-friendly multilayered systems demonstrate how CH–LNPs nanocomposites can be scaled for biodegradable paper packaging, replacing synthetic wax or polyethylene layers. Zhang et al. (158) used a deep eutectic solvent (DES)-assisted antisolvent method to prepare LNPs for CH film matrices. Their composite films exhibited ~40% higher tensile strength, significantly better UV-blocking, and enhanced radical scavenging capacity. Importantly, real-food applications demonstrated 10-day extended shelf life of refrigerated grass fish, confirming functional applicability for cold-chain packaging (158).

Another compelling approach was introduced by Vijayakumar et al. (178), who employed acid precipitation to generate LNPs of ~55 nm and incorporated them into CH films. With 15% LNPs loading, tensile strength and modulus increased by 86 and 93%, respectively, while water vapor transmission dropped by 32%. The films also achieved significant UV shielding and antioxidant improvements, critical for perishables sensitive to light and oxidation (178). Recent work by Abbadessa et al. (179) further explored layer-by-layer (LbL) assembly of lignin-based polymers with chitosan onto polyethylene substrates. While not fully biobased, this method demonstrated stepwise deposition of CH/LNPs bilayers, improving oxygen and water vapor barrier properties. This highlights CH’s versatility as both a standalone film matrix and a barrier-enhancing coating layer in hybrid designs (179).

Together, these studies demonstrate a consistent structure–property relationship: LNPs contribute to tighter polymer networks via hydrogen bonding, *π*–π interactions, and electrostatic attraction. These interactions reduce free volume, resulting in decreased gas/moisture diffusion and improved film integrity. Furthermore, the nanoscale dispersion of LNPs enables effective light scattering, enhancing UV blocking while contributing to opacity, a desirable trait in light-sensitive packaging. Despite these advances, key challenges remain. Achieving homogeneous LNPs dispersion, avoiding aggregation, and controlling interfacial compatibility require further optimization. Moreover, while antioxidant and antimicrobial activities are promising, their long-term migration behavior and safety profiles need evaluation for regulatory compliance. Thus, chitosan–LNPs nanocomposites are among the most promising fully biobased packaging candidates. They offer multifunctional enhancements, process compatibility, and effective food preservation, making them suitable for fresh produce, seafood, dairy, and bakery applications. With ongoing innovation in LNPs synthesis and film engineering, CH-based materials are poised to play a central role in next-generation sustainable packaging.

### 6.2 Starch-based systems (ST matrix)

Starch (ST), a low-cost, abundant, and renewable polysaccharide, has been widely studied as a sustainable alternative to petroleum-based films. Its natural biodegradability and film-forming ability make it attractive for food packaging. However, starch films inherently suffer from poor moisture resistance, low mechanical strength, and weak barrier properties, especially under humid conditions. To address these issues, incorporation of LNPs has been explored as a biocompatible, multifunctional strategy to reinforce starch matrices.

Firouzjaei et al. (180) developed starch-based nanocomposite films incorporating LNPs (1, 3, and 5% w/w) and cinnamaldehyde (CI), a natural antimicrobial agent. Their binary (ST–LNPs) and ternary (ST–LNPs–CI) films showed marked enhancement in physical and barrier properties. Water vapor permeability (WVP) decreased from 3.97 to 3.06 × 10^−1^⁰ g·s^−1^·m^−1^·Pa^−1^, and water solubility dropped from 53.00 to 19.76%—indicative of a denser, more hydrophobic polymer network. Tensile strength improved from 3.66 to 5.15 MPa with up to 3% LNPs. However, at 5% LNPs loading, a slight decline in tensile strength was observed, suggesting nanoparticle aggregation and phase separation. This underscores the importance of maintaining uniform LNPs dispersion to avoid forming defects that compromise membrane integrity—an issue echoed across multiple biopolymer matrices (180). Santhosh et al. (181) provided further evidence of LNPs–matrix synergy using a blend of litchi seed starch (LSS) and tamarind kernel xyloglucan (XG), reinforced with LNPs. Their LSS–XG–LNPs films exhibited a tensile strength of 14.83 MPa and an elastic modulus of 0.41 GPa, a significant improvement over neat LSS films. The water vapor permeability reduced to 5.63 × 10^−7^ g·m^−1^·s^−1^·Pa^−1^, and surface hydrophobicity increased (contact angle ~80°). The enhancement was attributed to strong hydrogen bonding between starch, XG, and LNPs, as well as the phenolic content of LNPs that conferred UV shielding and antioxidant protection. Application-wise, these films effectively extended the shelf life of bananas by minimizing weight loss and discoloration, validating real-world functionality (181).

Sun et al. (182) developed starch-based composite films using LNPs derived from bamboo via green fractionation. The 2% LNPs films showed optimal performance, with tensile strength increasing from 12.1 MPa (neat film) to 48.9 MPa, and modulus rising nearly 4-fold. FTIR confirmed hydrogen bonding, while SEM and AFM showed uniform dispersion at low LNPs content but agglomeration at 5%. Thermal stability improved by ~5–8 °C, and DPPH radical scavenging rose from 13.3 to 44.8% (2%) and 70.8% (5%). UV shielding was nearly complete at 2%, and oxygen permeability halved. The 2% LNPs film effectively delayed soybean oil oxidation, demonstrating strong barrier, UV-blocking, and antioxidant properties, making it a promising bio-based packaging solution (182).

Gai et al. (137) adopted a different approach, using LNPs with cationic starch as a coating material on paper substrates. The coating improved water resistance (Cobb value: 37.5 g·m^−2^), oil resistance (Kit rating: 9), and tensile strength (48.93 MPa), while lowering water vapor transmission more than sixfold. The use of LNPs provided superior interfacial adhesion and barrier formation. Though this system utilized paper as a structural base, the functional layer itself was built from starch and lignin, confirming their feasibility as green barrier alternatives to fluorinated or petroleum-based coatings (137).

Across these studies, a clear structure–property–function relationship emerges. LNPs act as nanoscale reinforcers that reduce polymer mobility, tighten chain packing, and introduce antioxidant and UV-resistant properties. Their interaction with starch matrices is governed by hydrogen bonding and phenolic *π*-interactions, leading to improved gas barrier, optical, and mechanical properties. However, above optimal LNPs loadings (typically >3–5%), performance degradation can occur due to aggregation, emphasizing the need for dispersion control and interfacial optimization. Therefore, starch–LNPs nanocomposites represent a scalable and eco-friendly solution for biodegradable packaging films, particularly in cold-chain, moisture-sensitive, and oxidation-prone applications. As part of the broader polysaccharide matrix class alongside chitosan, cellulose derivatives, and pectin, starch’s compatibility with LNPs makes it a key contributor to the ongoing replacement of fossil-based packaging with fully biobased systems. Future directions should focus on nanoparticle surface functionalization, co-polymer blends, and migration safety to enable industrial-scale translation.

### 6.3 Cellulose-based systems (CL matrix)

Cellulose, the most abundant natural polymer, offers significant potential as a sustainable and biodegradable substitute for petroleum-derived packaging materials. However, pure cellulose or regenerated cellulose (RC) films often suffer from poor moisture resistance, insufficient strength, and low durability. The integration of LNPs into cellulose-based systems presents an eco-friendly strategy to overcome these limitations by leveraging LNPs’ inherent rigidity, antioxidant potential, and UV-barrier properties. Recent studies have demonstrated a clear structure–property–function relationship between LNPs and cellulose matrices, positioning these nanocomposites as highly competitive alternatives to synthetic plastics.

Amini et al. (183) synthesized all-cellulose nanocomposite (ACNC) films using a green ionic liquid-assisted and ultrasound-modified approach, incorporating 3–7 wt% LNPs. Their films exhibited enhanced UV-blocking capacity (97% at 280 nm for 7% LNPs), and increased antioxidant activity (up to 68% over the control). Mechanical strength also improved with 3 and 5% LNPs additions, but began to decline at 7%, suggesting overloading-induced aggregation. The authors noted that excessive LNPs content could reduce film uniformity and diminish antibacterial activity (e.g., 63.88% reduction in *E. coli* inhibition at 7% LNPs). These findings emphasize the need to optimize nanoparticle concentration for balanced multifunctionality (183). Tian et al. (184) explored the incorporation of LNPs into bacterial cellulose (BC) during fermentation. While LNPs had negligible impact on BC productivity, they significantly retarded enzymatic biodegradability, enhancing the material’s stability in humid conditions. The extent of degradation delay was influenced by the source of technical lignin. For instance, BC films containing soda LNPs degraded only ~58 wt% under high enzyme load (5 mg·g^−1^ BCE), compared to ~97% for deep eutectic solvent (DES)-derived LNPs. This suggests that lignin–cellulose interactions can be tuned to modulate biodegradation kinetics, expanding the application scope to packaging scenarios requiring extended shelf life (184).

Tian et al. (185) synthesized high-strength regenerated cellulose (RC) films enriched with esterified lignin nanoparticles (ELNPs). With only 5% ELNPs, tensile strength soared to 110.4 MPa, and hydrophobicity improved dramatically (water contact angle: 103.6°; WVP: 1.127 × 10^−12^ g·cm·cm^−2^·s^−1^·Pa^−1^). Water absorption dropped to 36.6% at 120 min. These enhancements were attributed to the interfacial bonding between esterified LNPs and cellulose chains, which created a dense, cohesive film network. Moreover, the films exhibited complete biodegradation under soil conditions (12–30% moisture), satisfying environmental criteria for compostable packaging. The combination of mechanical robustness and environmental degradability underscores the potential of ELNPs–RC films as next-generation bioplastics (185). García-Fuentevilla et al. (186) introduced enzymatically polymerized LNPs into cellulose nanofiber (CNF) films via a laccase pretreatment. This method reduced particle size (6.8 ± 2.4 nm), increased molecular weight, and enhanced film performance. Films with 5% polymerized LNPs showed better thermal stability, UV shielding, and antioxidant and antibacterial activity than those with unmodified lignin. The enhanced properties are attributed to both the nanoparticle size reduction and the presence of conjugated phenolic structures, which reinforced the CNF matrix at both structural and functional levels. Importantly, elongation at break and film transparency also improved, indicating minimal trade-offs in flexibility and appearance (186).

Zhao et al. (187) conducted an in-depth mechanistic study on LNPs–cellulose reinforcement. They showed that LNPs melted during hot pressing and filled voids within the CNF matrix, leading to a dense, cross-linked structure. The resulting biocomposite achieved an exceptional tensile strength of 202 MPa, Young’s modulus of 9.55 GPa, and maximum degradation temperature of 375.1 °C. Surface analysis confirmed hydrogen bonding and self-assembly between CNFs and LNPs. Contact angle measurements (~70°) and 100% UV-blocking demonstrated the film’s dual hydrophobic and photoprotective functions. These results validate that LNPs can serve as both structural enhancers and functional additives, significantly advancing cellulose-based packaging technologies (187).

In all, these findings consistently demonstrate that LNPs–cellulose interactions play a pivotal role in shaping mechanical integrity, barrier functionality, and bioactivity of the resulting films. Mechanisms include hydrogen bonding, *π*–π stacking, esterification, and nanoparticle filling of microvoids, all contributing to compact, high-performance biocomposites. Notably, the nonlinear relationship between LNPs loading and film performance must be carefully managed, too little offers minimal benefit, too much causes aggregation and property deterioration. Positioned within the polysaccharide-based biopolymer class, cellulose and its derivatives form a cornerstone of biodegradable film development. Their compatibility with LNPs through multiple fabrication routes—whether by regeneration, fermentation, or mechanical dispersion—makes them strong candidates for commercial, fossil-free packaging solutions. Going forward, further efforts should aim at standardizing nanoparticle synthesis, improving dispersion control, and scaling up fabrication processes, while ensuring compliance with safety and regulatory requirements for food-contact materials.

### 6.4 Pectin-based systems (PC matrix)

Pectin is a plant-derived, anionic polysaccharide abundantly found in fruit peels and widely recognized for its excellent film-forming ability, edibility, biodegradability, and biocompatibility. As a representative of polysaccharide-based biodegradable materials, pectin is particularly well-positioned to replace petroleum-based plastics in active food packaging. However, native pectin films often suffer from poor mechanical strength, high water vapor permeability, and lack of bioactivity. Recent research has focused on the incorporation of LNPs into pectin matrices to overcome these limitations, not only by enhancing mechanical and barrier functions but also by imparting antioxidant and antimicrobial capabilities. The interactions between LNPs and the pectin network, via hydrogen bonding, entanglement, and electrostatic attractions, are central to these improvements, forming dense, nano-structured films with multifunctional behavior.

Zhang et al. (188) systematically investigated the effects of LNPs loading (1–3 wt%) on the performance of pectin-based films. Even at 1 wt%, the tensile strength (TS) of the composite increased by 67.33%, the water contact angle (WCA) by 48.83%, and the water vapor permeability (WVP) decreased by 25.30%. At the optimal concentration of 3 wt% LNPs, TS was enhanced by 164% and WCA by 56%, demonstrating a clear concentration-dependent correlation between LNPs addition and membrane functionality. The authors attributed this to the dense structural packing and strong interfacial compatibility between LNPs and the pectin chains, which significantly reduced free volume and diffusion pathways. Importantly, the composite films exhibited full UV-shielding capability, blocking nearly all UVC (200–275 nm) and UVB (275–320 nm) wavelengths, with considerable absorption in the UVA range as well. These films also showed marked improvements in bioactivity, with DPPH radical scavenging activity increasing up to 6.33-fold and antibacterial inhibition rates reaching 78.79% against *S. aureus* and 47.80% against *E. coli*. Such multifunctionality highlights the promise of pectin–LNPs films as active packaging for perishables, extending shelf life via both passive and active protection mechanisms (188). In a follow-up study, Zhang et al. (189) extended the concept by coating LNPs with polydopamine (PDA), forming hybrid LNPs@PDA particles that were uniformly dispersed in pectin matrices. Without using any conventional plasticizer, the resulting films demonstrated remarkable flexibility, with tensile strength reaching 35.76 MPa and WCA rising to 92.42°, indicative of enhanced hydrophobicity and mechanical resilience. The addition of LNPs@PDA also led to the formation of a nano-rough surface morphology (7.61–20.90 nm), which may further aid in controlling moisture diffusion. Notably, films with >5 wt% LNPs@PDA almost entirely blocked UVA, UVB, and UVC radiation, and retained UV-blocking capacity even after 24 h of continuous irradiation, confirming exceptional photostability. Beyond structural and protective enhancements, the LNPs@PDA-based films also demonstrated potent antioxidant and antimicrobial activity, thereby functioning as bioactive membranes. The authors proposed hydrogen bonding and *π*–π stacking as the dominant interaction mechanisms that support these improvements. Moreover, the shear-thinning property of the pectin–LNPs@PDA dispersion enabled its application via spray coating, suggesting promising utility as edible coatings or flexible surface films in food preservation (189).

Both studies emphasized not just the enhancement of film properties, but the mechanistic understanding of LNPs–pectin interactions, a focus that directly addresses reviewer concerns regarding weak correlation analysis in prior sections. The key mechanisms, hydrogen bonding, interfacial entanglement, and π–π interactions, are pivotal in tailoring film compactness, hydrophobicity, and biofunctionality. From a broader material perspective, pectin belongs to the polysaccharide-based biopolymer class, which is at the forefront of efforts to replace synthetic plastics with sustainable alternatives. Compared to protein-based or petroleum-derived systems, pectin–LNPs composites offer several distinct advantages: intrinsic edibility, compatibility with food-contact applications, tunable biodegradation, and a rich scope for chemical interaction with functional nanoparticles. The low required LNPs loadings (as low as 1–3 wt%) also support economic scalability and minimize any negative impact on film flexibility or visual transparency.

Hence, the integration of LNPs, either native or PDA-coated, into pectin matrices not only remedies the inherent weaknesses of pectin films but also introduces new functionalities that are highly desirable in modern food packaging. These developments position pectin–LNPs systems as strong candidates for replacing petroleum-based plastics in applications where biodegradability, UV resistance, and bioactivity are critical. Further research should explore scaling up production, regulatory compliance, and shelf-life performance under real-world conditions to facilitate industrial adoption.

### 6.5 Protein-based systems

Protein-based biopolymers such as gelatin and casein are widely recognized for their biodegradability, film-forming properties, and potential to replace petroleum-derived plastics in food packaging. However, their mechanical fragility, water sensitivity, and poor UV resistance necessitate functional enhancement. Recent studies have demonstrated that LNPs can effectively address these limitations by forming hydrogen-bonded networks with protein chains, improving structural integrity and bioactivity. Wang et al. (190) developed gelatin films enhanced with microwave-fractionated lignin (ML), achieving complete UV shielding and marked flame retardancy. The limiting oxygen index increased from <21% (combustible) in pure gelatin to 31.45% with ML addition, and further to 52.9% upon incorporating 20% ammonium polyphosphate (APP). Notably, the elongation at break increased over threefold, transforming the material from brittle to ultra-flexible while maintaining transparency. These results confirm the synergy between ML and gelatin matrices in tailoring both optical and mechanical behavior for safe packaging use (190).

In another study, Li et al. (191) fabricated casein (CA) films reinforced with LNPs derived from bamboo using an acid hydrotrope process. At 5 wt% LNPs, tensile strength improved by 220% (to 21.42 MPa) and modulus by a similar margin (354.9 MPa). These gains were attributed to templated hydrogen bonding and denser packing of protein chains. The nanocomposite films also exhibited nearly complete UV absorption (200–400 nm), reduced water solubility (from 31.7 to 24.8%), and enhanced antioxidant and antibacterial properties. Even after three recasting cycles, mechanical strength remained adequate, highlighting reprocessability. Additionally, soil burial tests showed >100% mass loss within 45 days, confirming excellent biodegradability (191). Thus, these examples underscore the pivotal role of LNPs in boosting the performance and sustainability of protein-based films, positioning them as viable bioplastic alternatives. Their ability to improve tensile strength, UV resistance, and barrier function through specific interactions with protein matrices marks a key advancement in green packaging design.

### 6.6 Polyvinyl alcohol-based systems

Although polyvinyl alcohol (PVA) is a petroleum-derived synthetic polymer, it is widely classified as a biodegradable material under aerobic industrial conditions and is frequently studied in the context of sustainable packaging. In this review, the inclusion of PVA aligns with our broader objective: to evaluate LNP incorporation into biodegradable food packaging films, regardless of the polymer’s origin. PVA-based systems serve as an important transitional platform and model matrix for exploring nanoparticle–polymer interactions due to their excellent film-forming properties, transparency, and compatibility with LNPs. The insights derived from PVA–LNP systems, especially regarding mechanical reinforcement, UV shielding, and antimicrobial behavior, can inform the design of future fully bio-based materials. However, their synthetic origin and limited compostability mean they are best viewed as intermediate solutions rather than long-term sustainable replacements. Nevertheless, PVA-based films, like many biopolymer-based systems, suffer from drawbacks such as limited UV-blocking capability, moisture sensitivity, and a lack of inherent antioxidant or antimicrobial activity. To address these limitations and enhance the functional profile of PVA films, the incorporation of LNPs has emerged as a highly effective strategy, enabling the creation of active packaging systems with improved mechanical properties, barrier performance, and bioactive functions. While PVA is not fully bio-based, its use as a biodegradable matrix for bio-derived LNPs represents a transitional solution toward reducing reliance on conventional petroleum-based membranes. Compared to standard fossil-derived films like polyethylene or polystyrene, these PVA–LNPs composites offer greater environmental compatibility and multifunctional benefits.

Zhang et al. (192) developed PVA/LNPs nanomicelle films where lignin nanomicelles (LNM) were homogeneously dispersed, forming strong hydrogen bonds with PVA. With just 5 wt% LNM, the water vapor transmission rate (WVTR) decreased by 189% compared to pure PVA, indicating enhanced barrier functionality. UV–Vis spectroscopy showed near-total UV absorption below 400 nm, providing UV-shielding. Mechanical testing revealed improved tensile strength and toughness. The films maintained flexibility and transparency, making them suitable for packaging applications requiring optical clarity. The correlation between nanomicelle dispersion and moisture barrier enhancement was clearly established, making this study a foundational example of structure–property enhancement through hydrogen bonding networks (192). Li et al. (193) utilized a green DES system to extract LNPs from walnut shells and integrated them into a PVA matrix with *ε*-polylysine (ε-PL). The 203 nm LNPs (*ζ*-potential −28.29 mV) exhibited enhanced antioxidant activity. Electrostatic and non-covalent interactions between LNPs and ε-PL improved tensile strength to 33.32 MPa and UV-blocking to 90%. Antimicrobial assays showed 60% inhibition, attributed to phenolic hydroxyl release. The film reduced mold growth in walnut storage trials. Unlike previous abstract-level reviews, this study offers comprehensive material–application linkage: morphological features, radical scavenging capacity, and food preservation performance were all quantified, supporting lignin’s active role. It exemplifies the shift from passive barrier materials to multifunctional smart films (193).

Kirar et al. (194) produced PVA–PEG films embedded with wheat-straw-derived LNPs and lignin–copper oxide nanohybrids. The resulting PVA–PEG–PNSL–L@CuONPs films demonstrated antimicrobial activity exceeding 5-log reductions against *E. coli*, *B. megaterium*, and *C. albicans*. UV protection was also validated, though quantitative transmission data was not provided. Notably, the films maintained optical transparency and flexibility, showcasing lignin’s dual role as a bioactive and structural enhancer. The nanocomposites’ functionality stemmed from synergistic antimicrobial mechanisms (CuO ion release and lignin phenolics) and dense LNPs networks improving barrier properties. This study advances the use of LNPs not merely as fillers but as active antimicrobial agents within hybrid membrane architectures (194).

Zheng et al. (156) synthesized LNPs using a DES-based antisolvent method and incorporated them into PVA films. The resulting LNPs (586–848 nm) displayed uniform dispersion and strong hydrogen bonding with the PVA matrix. Mechanical testing revealed significant improvements in tensile strength and thermal stability. The hydrophobicity of the films increased due to the *π*–π stacking of lignin’s aromatic rings. Importantly, the films blocked nearly all UV light below 400 nm and exhibited potent antibacterial activity against *S. aureus* and *E. coli*. The authors linked particle stability (*ζ*-potential), interaction chemistry, and end-use performance, providing a clear structure–function–application pathway lacking in earlier reports (156). Zeng et al. (195) engineered multifunctional PVA films incorporating tannin acid (TA) and LNPs loaded with potassium sorbate (LNPs@PS). Optimal formulation (3% LNPs@PS + 5% TA) achieved tensile strength of 74.5 MPa, over 2.5 times that of pure PVA. Water vapor and oxygen permeability decreased by 47 and 112%, respectively. Films exhibited strong antibacterial action and UV-blocking. Slow-release of PS prolonged strawberry shelf-life by 3 days. Unlike generic summaries, this study emphasized lignin’s encapsulation and release kinetics, and the synergistic mechanical reinforcement from TA–PVA–LNPs interactions. It exemplifies LNPs as both physical enhancers and active carriers in intelligent packaging (195).

**Figure 5:**
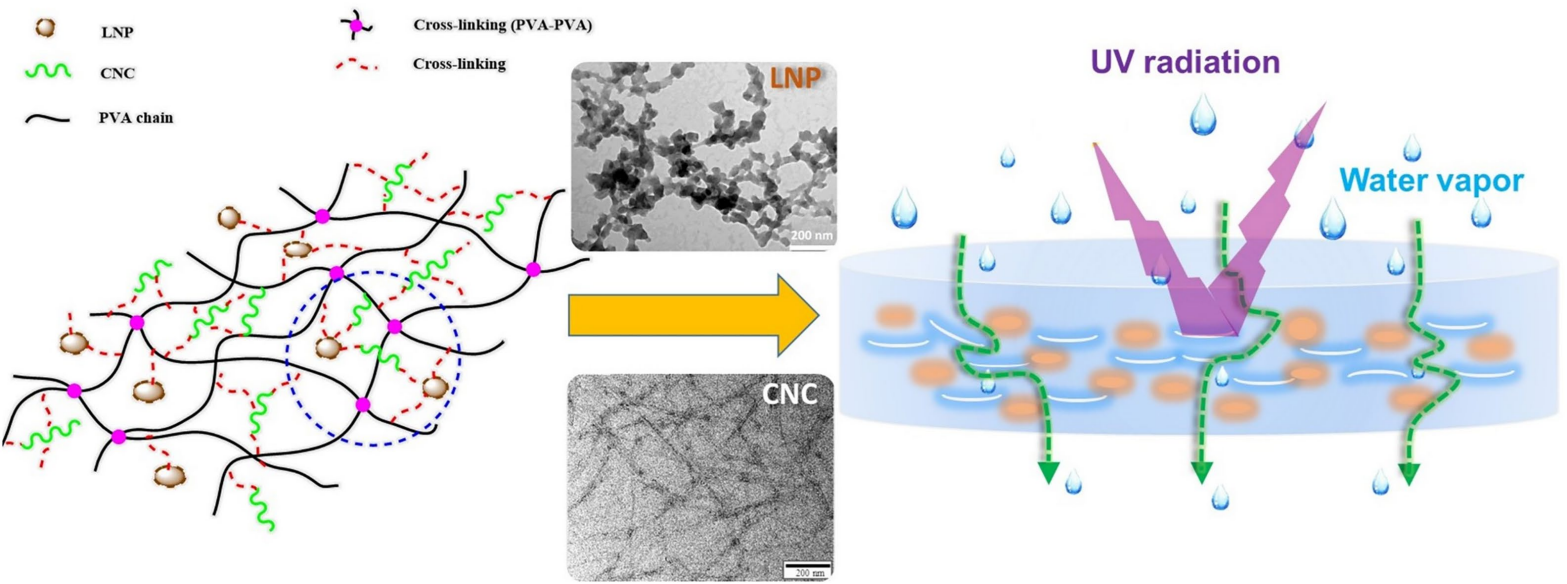
Schematic representation of the ternary PLA nanocomposite structure incorporating cellulose nanocrystals (CNCs) and lignin nanoparticles (LNPs). The synergy between CNCs and LNPs enhances mechanical strength, water vapor barrier properties, UV shielding, and antibacterial activity of the PLA films. Reproduced from (196), licensed under CC BY 4.0.

Yang et al. (196) formulated glutaraldehyde-crosslinked PVA nanocomposites with CNCs and LNPs (Figure 5). Thermal stability improved due to covalent crosslinking and strong hydrogen bonding. The inclusion of 1 wt% LNPs and 2 wt% CNC increased tensile strength from 26 MPa (neat PVA) to 35.4 MPa. Radical scavenging assays confirmed high antioxidant capacity, while water vapor permeability decreased. Importantly, the interplay between CNC crystallinity and LNPs amorphous structure led to balanced ductility and stiffness. The study demonstrated the sacrificial hydrogen bond mechanism, where LNPs absorb stress and prevent crack propagation, clarifying how lignin contributes at the molecular level to membrane resilience (196). Yang et al. (197) fabricated binary and ternary nanocomposites based on PVA, chitosan, and LNPs (1–3 wt%). LNPs improved tensile strength and Young’s modulus, with crystallinity enhancement confirmed by DSC. SEM revealed uniform dispersion and absence of phase separation. Antibacterial tests showed efficacy against *Erwinia carotovora* and *Xanthomonas arboricola*, pathogens relevant to fresh produce. The combination of LNPs and chitosan resulted in synergistic antioxidant activity, expanding application potential to both food and biomedical packaging. The study established lignin as both a mechanical and functional additive in multi-polymer systems, correcting the misconception of its role as a passive filler (197).

He et al. (198) investigated citric acid-modified LNPs (MLNPs) blended with PVA via solvent casting. SEM confirmed homogeneous dispersion with no aggregation. Contact angle and swelling tests showed increased hydrophobicity and dimensional stability in MLNPs-based films. Antioxidant activity outperformed unmodified LNPs, while UV shielding slightly declined. The trade-off between light transmittance and radical scavenging was discussed. The study highlighted that surface-functionalized lignin influences interfacial bonding, diffusion resistance, and water adsorption. By balancing transparency with bioactivity, this work deepens understanding of tunable lignin design for custom performance in membrane applications (198).

**Figure 6:**
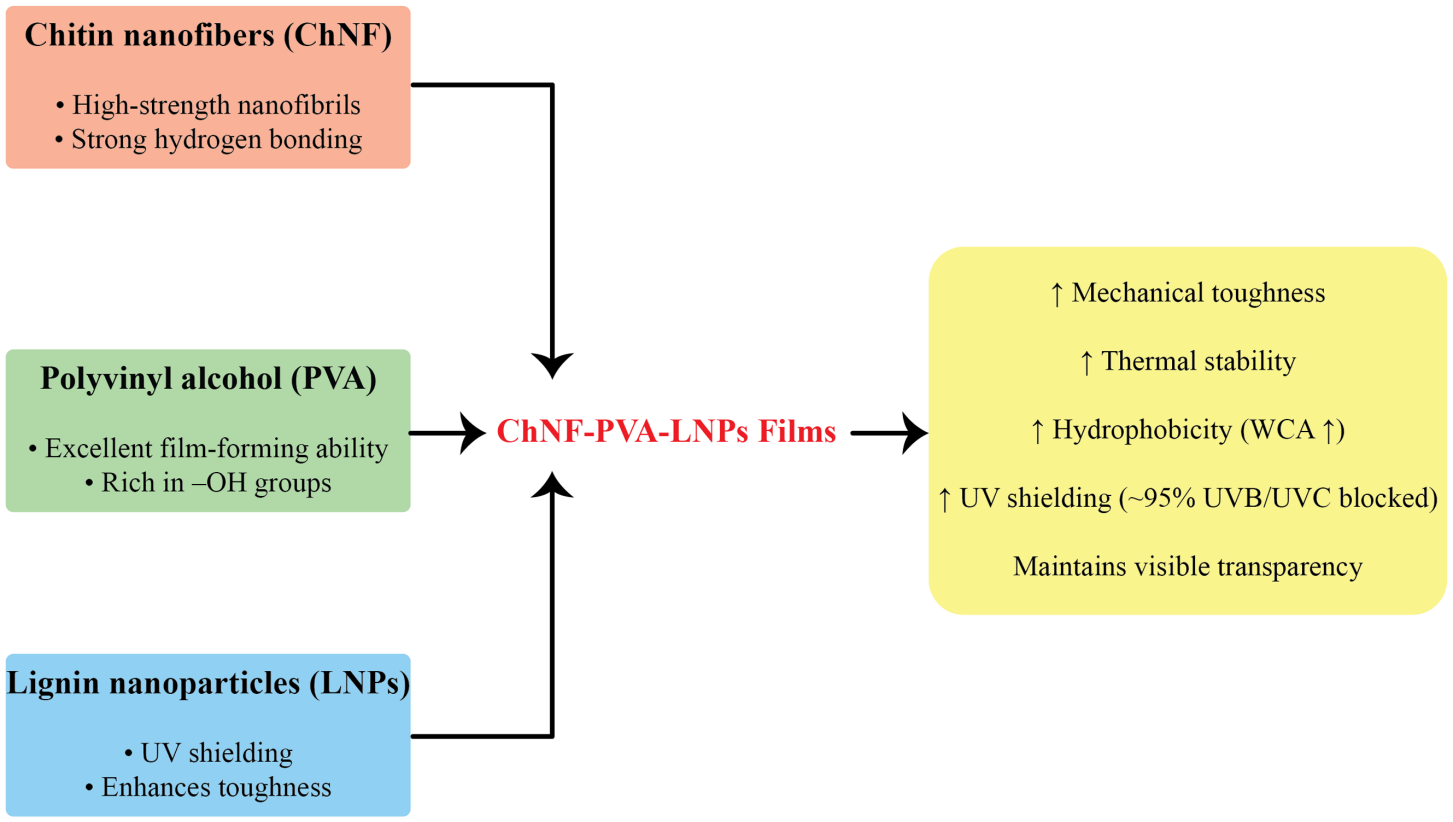
Schematic illustration of the preparation of ternary PVA nanocomposite films incorporating chitin nanofibers (ChNF) and lignin nanoparticles (LNPs). The synergistic addition of ChNF and LNPs enhances mechanical toughness, UV shielding (>95% UVB/UVC blocking), thermal stability, and surface hydrophobicity while maintaining high visible light transmittance.

Both variants (P-GA-3LNPs and P-CA-3LNPs) exhibited zero UV-B/UV-C transmittance. TGA showed enhanced thermal stability. Tensile strength improved from 26 MPa (PVA) to 38.1 and 32.7 MPa for GA and CA crosslinked films, respectively. Elongation at break remained ~230%. P-GA-3LNPs displayed superior antibacterial activity. Fluorescence imaging after shrimp packaging confirmed microbial inhibition under UV. The data reinforced that lignin’s function varies with crosslinker type and degree of network formation, offering insights into film flexibility versus barrier optimization. Moreover, Zhou et al. (199) developed ternary PVA nanocomposite films by incorporating both chitin nanofibers (ChNF) and lignin nanoparticles (Figure 6). The addition of 5% ChNF and 1% LNPs significantly enhanced the mechanical toughness, thermal stability, surface hydrophobicity, and UV shielding of the PVA films, achieving up to 95% UVB and UVC blocking efficiency while maintaining transparency. These properties are particularly advantageous for sensitive food products that are prone to UV-induced degradation. By leveraging the synergy between ChNF and LNPs, the films not only exhibited superior structural properties but also provided improved functional performance compared to binary PVA blends.

Tian et al. (200) compared hollow (HLNPs) and solid (SLNPs) lignin nanoparticles synthesized from eucalyptus and willow using organosolv pretreatment. Films with SLNPs reached tensile strength of 132 MPa, while HLNPs enabled 324% elongation at break. Water contact angle exceeded 90° in both types, confirming hydrophobicity. The study introduced the “room-like” network hypothesis, where HLNPs increase free volume and toughness. This comparative morphology-based insight is critical for designing lignin structures tailored to desired membrane mechanics. The research bridges nanoparticle morphology with macroscopic performance in a rare yet practical way (200). Xu et al. (201) synthesized LMNPs using *γ*-valerolactone and optimized parameters (stirring, solvent ratio, lignin concentration) to control nanoparticle size (157–442 nm). The resulting PVA/LMNP films exhibited enhanced UV shielding, thermal stability, hydrophobicity, and mechanical integrity. PVA films showed significant antioxidant activity and stability over a pH range of 4–12. The study emphasized process–property correlation by linking synthesis control (e.g., dropping rate) with final film performance. It also reinforced the value of tailoring lignin structure–aggregation behavior to fine-tune multifunctionality in packaging applications, beyond simple performance reporting (201).

Despite being petroleum-derived, PVA serves as a valuable model matrix for studying LNPs interactions due to its transparency, processability, and hydrophilic character. The reviewed studies show that the addition of LNPs, particularly those engineered via DES, microwave, or acid-hydrotrope methods, induces significant improvements in tensile strength, thermal stability, UV resistance, and barrier performance. These enhancements arise from hydrogen bonding, aromatic *π*–π stacking, nanoparticle-induced crystallinity, and in some cases, covalent crosslinking, all of which reduce free volume and enhance structural integrity. Crucially, the films also exhibit functional bioactivity, such as antioxidant and antimicrobial behavior, with certain systems achieving this without added plasticizers or preservatives. These properties, particularly when combined with slow-release antimicrobial agents or crosslinking agents, enable PVA–LNPs systems to simulate “active packaging” scenarios applicable to perishables like berries, seafood, and baked goods. However, the sustainability of PVA remains questionable due to its synthetic origin. While it can be partially biodegraded under industrial conditions, it is not aligned with the broader push for natural, compostable food-contact films. Thus, PVA–LNPs composites are best viewed as transitional or hybrid systems, ideal for understanding nanocomposite design rules and benchmarking biopolymer alternatives, rather than as ultimate solutions to plastic pollution. Future research should prioritize transferring these design principles, particularly structure–function correlations and surface modification strategies, to fully biobased matrices such as starch, pectin, and cellulose derivatives for true sustainability.

### 6.7 Polylactic acid based systems

Polylactic acid (PLA), a biodegradable polyester derived from renewable resources such as corn starch and sugarcane, is widely used in food packaging due to its mechanical strength, transparency, and processability. However, its poor UV shielding, limited antioxidant activity, and brittleness restrict broader applications. LNPs offer a sustainable pathway to overcome these limitations, serving as bioactive fillers that enhance both functionality and environmental compatibility.

Yang et al. (202) developed ternary nanocomposite films by combining PLA with cellulose nanocrystals (CNCs) and LNPs at 1 and 3% loadings, respectively. Both neat and glycidyl methacrylate-grafted PLA were used as matrices. The combined addition of CNC and LNPs produced a synergistic effect, enhancing crystallinity, tensile strength, and Young’s modulus beyond binary systems. Notably, UV–Vis analyses confirmed enhanced UV blocking and transparency. The ternary films also displayed antibacterial effects against *Pseudomonas syringae*, highlighting their value for active packaging. The study emphasized that LNPs not only reinforce mechanical and optical properties but also impart bioactivity. The uniform dispersion of both lignocellulosic nanofillers facilitated matrix–particle interaction, creating ordered domains that increased barrier function while maintaining biodegradability and compostability (202). Cavallo et al. (203) examined PLA films incorporating pristine, citric-acid-modified (caLNPs), and acetylated (aLNPs) lignin nanoparticles at 1 and 3% loadings. All films demonstrated enhanced UV protection, antioxidant capacity, and antibacterial activity. While chemical modifications slightly decreased antioxidant and UV shielding performance, they improved nanoparticle dispersion, reduced color intensity, and enhanced ductility. Migration tests confirmed their safety in food contact conditions, and composting simulations demonstrated full disintegration within acceptable timeframes. These results underscore the dual benefits of functional and aesthetic improvements via surface modification of LNPs. The balance between nanoparticle performance and film appearance is particularly relevant in consumer-facing packaging markets (203).

Boarino et al. (170) addressed the challenge of LNPs aggregation in PLA matrices by grafting PLA chains onto lignin nanoparticles via organocatalyzed lactide ring-opening polymerization. This modification allowed for uniform dispersion of LNPs up to 10 wt%, eliminating aggregation and enabling consistent optical clarity. As little as 1 wt% PLA-grafted LNPs effectively blocked 280 nm UV light while retaining visible transparency. The films also exhibited significantly higher antioxidant activity compared to unmodified PLA or PLA–LNPs composites, confirming that surface engineering of LNPs enhances both their functionality and compatibility with the matrix. The study highlights the necessity of nanoparticle tailoring for advanced PLA applications where both visual and active properties are critical (170). Daassi et al. (164) employed an electrospray method to fabricate LNPs from rice husk lignin and integrate them into PLA films. By optimizing lignin concentration, flow rate, voltage, and tip-to-collector distance, they produced spherical LNPs (~260 nm, −35.2 mV zeta potential) with excellent dispersion. Films containing PLA-grafted LNPs showed up to 4-fold increase in elongation at break, with UV transmittance reduced from 58.7 to 1.1%. Antioxidant activity was boosted more than 12-fold. These improvements were attributed to better interfacial compatibility and nanoparticle dispersion. The grafting process enhanced the matrix–filler interaction, leading to tighter networks and efficient light-blocking without sacrificing transparency, critical for visually appealing food packaging. This study reinforces the potential of functionalized LNPs in strengthening PLA film integrity while adding active protection (164).

Across all studies, LNPs act as multifunctional enhancers in PLA matrices, improving UV shielding, mechanical resilience, antioxidant capacity, and biodegradability. The key determinant of success lies in controlling LNPs morphology and interfacial compatibility. Grafted or chemically modified LNPs demonstrate superior dispersion, leading to homogenous membranes with fewer defects, tighter packing, and reduced optical haze. Importantly, despite PLA being biobased, its partial reliance on industrially processed monomers and limited barrier function justifies its reinforcement with LNPs derived from lignocellulosic waste. Thus, the integration of tailored LNPs into PLA aligns both with sustainability goals and the functional demands of modern active packaging.

### 6.8 Polybutylene succinate- and polybutylene adipate terephthalate-based systems

PBS and PBAT are aliphatic polyesters regarded as potential biodegradable alternatives to petroleum-based plastics due to their processability, biodegradability, and film-forming capability. The integration of LNPs into these polymers offers a sustainable route to enhance physicochemical and functional properties while embedding antioxidant and antimicrobial functionalities. Moe et al. (204) incorporated LNPs into PBS to develop antifungal packaging films for extending the shelf life of bread. At 0.5% (w/v), LNPs achieved fungal growth inhibition (FGI) rates of 51.89 and 55.94% against *Aspergillus niger* and *Penicillium* spp., respectively. When combined with 5% cinnamaldehyde (CIN), PBS films containing 1% (w/w) LNPs showed enhanced antifungal efficacy, particularly against *Penicillium*. Importantly, the addition of LNPs improved water contact angle and barrier properties without significantly affecting tensile strength, Tg, or Tm. Bread packed in PBS/LNPs/CIN films exhibited the lowest yeast and mold count (YMC < 1.0 log CFU/g), maintaining microbial stability over 14 days. This indicates that LNPs improve shelf-life performance by reinforcing barrier functions and synergizing with natural antimicrobials. The incorporation of 1% (w/w) LNPs improved thermal stability (Td increase from 354.1 to 364.7 °C) and Tg (−39.1 to −35.7 °C), without compromising melting point or crystallinity. Tensile strength increased to 35.6 MPa compared to the neat PBS film, while water and oxygen vapor permeability decreased, enhancing film compactness. FTIR and FE-SEM revealed strong interactions and homogeneous distribution of LNPs and thymol. The ternary composite (PBS + 1% LNPs + 10% thymol) effectively inhibited *Colletotrichum gloeosporioides* and *Lasiodiplodia theobromae* both *in vitro* and in mango packaging trials. This study underscores the multifunctional enhancement of PBS films via LNPs-mediated synergy with bioactive agents (204).

**Figure 7:**
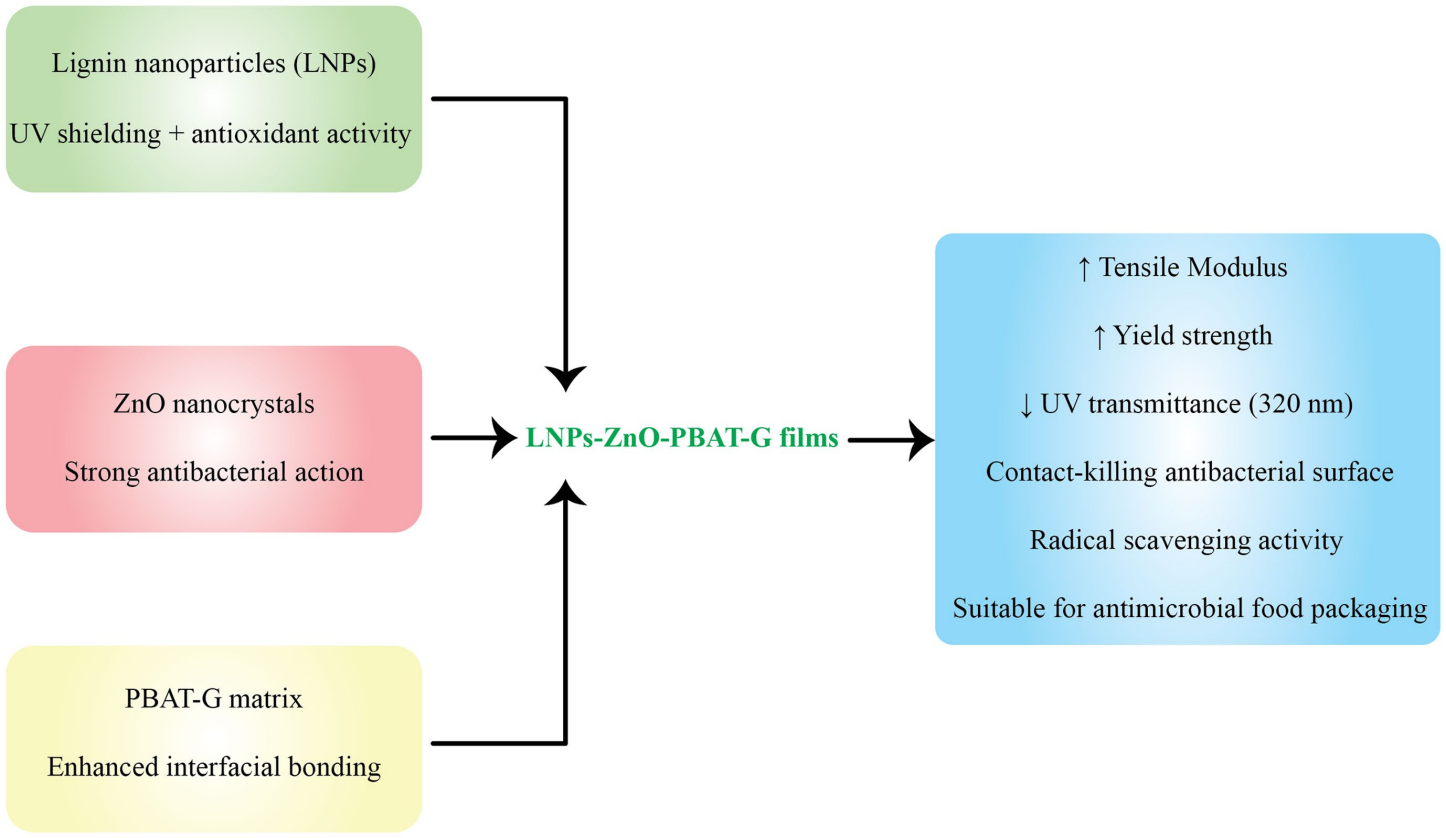
Schematic illustration of the preparation of PBAT composite films incorporating lignin–zinc oxide (LZn) hybrids. The films exhibit improved UV shielding, antibacterial activity against *E. coli* and *S. aureus*, and enhanced mechanical performance, highlighting their potential for active food packaging applications.

Nuamduang et al. (205) developed PBS/LNPs/trans-cinnamaldehyde (CN) composites via blown film extrusion to improve mango preservation. The study showed that LNPs facilitated higher retention of CN and improved film homogeneity. PBS/LNPs/CN films exhibited reduced diffusion coefficient, enhanced barrier properties, and effective inhibition of *C. gloeosporioides* in vitro and *in vivo*. SEM confirmed a more compact surface structure, and thermal analysis showed stable transitions. The composite films maintained mango quality during storage and extended shelf life by controlling fungal growth. The role of LNPs in sustained CN release was critical in maintaining antifungal activity, highlighting their function as nano-reservoirs within the polymer matrix (205). Xiao et al. (206) introduced lignin–ZnO (LZn) hybrid particles into PBAT via a hydrothermal synthesis followed by grafting onto glyceryl methacrylate-modified PBAT (PBAT-G) (Figure 7). Among tested variants, PBAT-G-3LZn showed a 35.2% increase in tensile modulus and 28.6% higher yield strength. The LZn hybrids significantly outperformed neat LNPs in antibacterial activity, lowering *E. coli* and *S. aureus* survival rates to 9 and 49%, respectively. UV transmittance at 320 nm dropped to 0.16%, while radical scavenging activity reached 11.6%. Zn^2+^ migration remained within safe limits (3.3 μg/L). The composite exhibited strong contact-killing effects, making it a viable option for antimicrobial food packaging with robust physicochemical integrity (206).

Venkatesan et al. (207) prepared PBAT composites with lignin–TiO₂ nanoparticles (L-TiO₂) using melt processing and hot pressing. The films displayed improved crystallinity, thermal stability, and tensile strength, increasing from 24.3 MPa (neat PBAT) to 47.0 MPa with 5% L-TiO₂. SEM and FTIR confirmed uniform nanoparticle dispersion and strong interfacial compatibility. Barrier properties were significantly improved, and the composite film showed broad-spectrum antibacterial activity. Packaging trials with strawberries demonstrated superior preservation compared to commercial polyethylene films. The study highlights the role of L-TiO₂ in reinforcing PBAT films both structurally and functionally, leveraging lignin’s compatibility and TiO₂‘s antimicrobial features (207). Marcoaldi et al. (208) investigated electrospun PHBV films embedded with LNPs synthesized via a green, scalable method. Although not PBAT or PBS, this PHBV-based study offers valuable insight into LNPs dispersion and structure–function relationships. PHBV/LNPs films maintained mechanical integrity and achieved 90% DPPH inhibition even after 20 days, while reducing *S. aureus* counts by ~10-fold. Oxygen permeability decreased by up to 10 × compared to neat PHBV. These outcomes reinforce that uniformly distributed LNPs can significantly enhance antioxidant, antimicrobial, and barrier functions in biopolyester matrices without the need for chemical modification (208). Collectively, these studies confirm that LNPs are not merely passive fillers but active nanostructured reinforcers that interact with PBS/PBAT matrices to enhance thermal, mechanical, barrier, and bioactive properties. Their synergy with bioactive agents like cinnamaldehyde or thymol further amplifies antimicrobial efficacy, making them ideal for active food packaging. Consistent with reviewer comments, this section demonstrates detailed structure–property-performance correlations, moving beyond abstract-level summaries. LNPs contribute to shelf-life extension, reduce environmental burden, and offer a biocompatible pathway toward replacing petroleum-based materials in commercial packaging applications.

### 6.9 Next,-generation substrates and structural engineering strategies in LNPs-based food packaging

While much of the recent literature has focused on integrating LNPs into traditional biopolymer matrices, such as chitosan, starch, cellulose, PLA, and PBS, emerging strategies now seek to redefine both the substrate architecture and the structural design of the nanoparticles themselves. These novel approaches aim to surpass the performance limits of conventional composites by introducing new platforms, processing methods, and hybrid mechanisms. This section highlights such developments, focusing on underexplored matrices, advanced structural tuning of LNPs, and unconventional formats like layer-by-layer coatings and electrospun membranes.

One emerging avenue involves the use of non-traditional biopolymer matrices such as macroalgae-derived films. Alfatah and Abdul Khalil (209) introduced a strategy that valorizes coconut fiber waste to produce LNPs and incorporates them into macroalgae-based films. With only 6 wt% LNPs, the resulting biofilms achieved a tensile strength enhancement of over 60% and a water contact angle exceeding 100°, demonstrating significantly improved mechanical robustness and hydrophobicity. These composites retained high transparency and showed marked antioxidant activity. The compatibility of lignin with marine biopolymers opens new avenues for edible and biodegradable packaging, particularly in coastal food systems where seaweed or algae resources are abundant and underutilized (209). In another notable departure from mainstream materials, poly(3-hydroxybutyrate-co-3-hydroxyvalerate) (PHBV) has emerged as a viable matrix for LNPs integration. Marcoaldi et al. (208) electrospun PHBV fibers incorporating green-synthesized LNPs and annealed them into continuous films. Compared to PHBV films loaded with unmodified kraft lignin, the LNPs-based composites showed superior dispersion, higher antioxidant activity (~90% DPPH inhibition), and an order-of-magnitude improvement in oxygen barrier performance. These outcomes were achieved without compromising the mechanical integrity of PHBV, making the material suitable for high-barrier and compostable packaging applications. Notably, this system maintained mechanical and optical clarity without requiring chemical surface modification of the nanoparticles.

Advancements have also been made in the synthesis and structural engineering of LNPs themselves. Daassi et al. (164) optimized electrospray parameters, including lignin concentration, voltage, and flow rate, to create uniform LNPs with tunable diameters (~260 nm), high zeta potentials (−35 mV), and excellent dispersion in PLA matrices. These electrosprayed LNPs exhibited enhanced antioxidant activity and UV-barrier function while minimizing optical haze. Unlike conventional antisolvent methods, electrospray fabrication allows precise morphological control, enabling the production of highly monodisperse, application-specific LNPs. Films prepared with PLA-grafted LNPs demonstrated greater elongation at break, higher antioxidant potential (12 × that of neat PLA), and near-complete UV-blocking in the 200–320 nm range—all while preserving visible transparency. In a separate development, enzyme-polymerized LNPs have been employed to modify cellulose nanofiber (CNF) membranes. García-Fuentevilla et al. (186) demonstrated that enzymatically polymerized LNPs (via laccase treatment) achieved a ~ 6.8 nm particle size and improved thermal stability, UV shielding, and antibacterial efficacy relative to unmodified LNPs. Their integration into CNF matrices produced films with increased elongation at break and superior free radical scavenging, without sacrificing clarity or flexibility. These results highlight the role of molecular architecture, particularly conjugated phenolic networks, in modulating nanoparticle–matrix interactions and enhancing membrane resilience (186).

Another frontier involves layer-by-layer (LbL) assembly of LNPs onto synthetic or paper substrates, enabling the transformation of petroleum-based materials into hybrid bioactive films. Abbadessa et al. (179) constructed multilayer coatings of a lignin-derived polymer (EH) with polyethylenimine (PEI) or chitosan (CH) on polyethylene (PE) films. Using up to 20 bilayers, they achieved substantial reductions in oxygen and water vapor permeability and enhanced light barrier properties. QCM-D and SPAR techniques confirmed stepwise deposition and stable polyelectrolyte complex formation. The LbL approach enables precision control over film thickness, composition, and function, offering a scalable route for upgrading commodity plastics into partial bio-based systems with improved sustainability profiles (179).

A complementary innovation is the sprayability of LNPs-based dispersions. Zhang e al. (189) demonstrated that polydopamine-coated LNPs (LNPs@PDA) could be homogeneously suspended in various polysaccharide matrices with shear-thinning behavior, allowing application via common spray nozzles. This feature enables on-site formation of active coatings on fresh produce, food wraps, or other surfaces. The sprayed films retained high mechanical strength (~35 MPa), exhibited full UV shielding across UVA–UVC spectra, and provided robust antibacterial and antioxidant functionality even without added plasticizers. The ability to deploy LNPs in a spray-on format dramatically expands their applicability in minimally processed or decentralized food packaging contexts (189). These strategies are united by a shared emphasis on modularity and multifunctionality. Whether through electrospray precision, enzymatic polymerization, or nanoscale coating design, each method pushes LNPs-based packaging toward adaptive performance in real-world settings. Moreover, many of these systems show long-term bioactivity retention, enhanced photostability, and controlled degradation, offering advantages over conventional bulk fillers or volatile antimicrobial agents.

Thus, next-generation LNPs-based food packaging is no longer confined to improving conventional matrices. By rethinking both the substrate and nanoparticle architecture, researchers are developing systems that not only surpass current barrier and mechanical benchmarks but also offer functionality such as tunable release, sprayability, recyclability, and extended durability. A frontier yet to be fully explored in LNP-based systems is the development of smart response films, materials capable of responding to external stimuli such as pH, temperature, gases, or microbial activity. While some studies mentioned above exhibit passive controlled-release behavior [e.g., LNPs@PS (195) or LNPs–CIN systems (204, 205)], truly stimuli-responsive mechanisms, such as colorimetric shifts, freshness indicators, or signal-generating membranes, have not been widely implemented with LNPs. Given the redox-active, phenolic-rich structure of lignin, future research could harness its reactive chemical groups to develop intelligent films that change color in response to spoilage gases, release antimicrobials upon humidity changes, or provide visual indicators of contamination. Moreover, lignin’s UV-absorbing and radical-scavenging properties make it a promising scaffold for responsive dyes, electrochromic elements, or enzyme-linked indicators. Integrating LNPs with responsive polymers, natural dyes (e.g., anthocyanins), or gas-sensing molecules represents an important direction for enabling next-generation smart packaging. These innovations point toward a future where LNPs are not merely additives but core design elements in sustainable, high-performance food packaging ecosystems.

## 7 Limitations

Despite remarkable advancements in LNPs-based food packaging, several critical limitations continue to hinder their transition from laboratory-scale innovation to industrial application. A major bottleneck lies in the scalability and reproducibility of LNPs synthesis. Current methods, such as antisolvent precipitation, ultrasonication, or dialysis, often suffer from batch-to-batch variability, especially when lignin is sourced from different biomass feedstocks or extracted via chemically divergent methods (e.g., kraft vs. organosolv vs. DES). The physicochemical characteristics of lignin, molecular weight, hydroxyl group content, degree of condensation, and presence of impurities, can vary widely, leading to inconsistent nanoparticle morphology, size distribution, and surface functionality. This heterogeneity undermines process control and complicates efforts to establish standardized protocols essential for regulatory approval and industrial scaling.

Another pressing issue involves the stability and uniformity of LNPs within biopolymer matrices. While LNPs can enhance mechanical and barrier properties, their tendency to aggregate at concentrations above 3–5 wt% can result in heterogeneous dispersion, phase separation, and even microstructural defects in the resulting films. These phenomena compromise not only the mechanical integrity and transparency of the packaging materials but also the reproducibility of functional properties such as antioxidant activity or UV blocking. Although surface modification of LNPs (e.g., with citric acid, polydopamine, or grafted PLA chains) has been proposed to improve compatibility, such strategies introduce added synthesis steps, material costs, and sometimes reliance on non-renewable chemical agents—thereby diminishing the overall sustainability profile.

A further limitation lies in the uncertainty around long-term safety and regulatory compliance. While lignin is generally recognized as safe in bulk form, the behavior of lignin-derived nanoparticles under food-contact conditions is not fully understood. There is insufficient toxicological data on their potential cytotoxicity, genotoxicity, or interactions with digestive enzymes, especially after prolonged storage or under thermal or mechanical stress. Moreover, migration behavior, the extent to which LNPs or phenolic degradation products might leach into food under various storage conditions (e.g., acidic pH, fatty content, UV exposure), has not been systematically assessed. These gaps make it challenging to meet the stringent food contact safety requirements outlined by regulatory bodies such as the U. S. Food and Drug Administration (FDA) or the European Food Safety Authority (EFSA).

In addition, the recyclability and biodegradability of LNPs-enhanced biopolymer films have not been sufficiently validated under real-world waste management scenarios. While individual components (e.g., chitosan, PLA) are biodegradable, the influence of LNPs inclusion on compostability, microbial degradation rates, or fragmentation behavior remains underexplored. Furthermore, few studies have evaluated whether LNPs retain their antioxidant or antimicrobial functionality after multiple processing or recycling cycles, an important consideration for packaging applications intended to align with circular economy goals. Without robust data on end-of-life behavior, including whether LNPs accumulate in the environment or interfere with organic recycling streams, claims of sustainability remain speculative.

Lastly, economic considerations pose significant challenges. While LNPs offer performance enhancements, their production, particularly through greener but more expensive methods like DES extraction or electrospray synthesis, may not yet be cost-competitive with conventional additives or synthetic nanoparticles (e.g., TiO₂, ZnO). Until processing routes are optimized for scalability, yield, and cost-effectiveness, widespread industrial adoption will remain limited. Thus, addressing these multidimensional limitations, technical, environmental, regulatory, and economic, is imperative to unlock the full potential of LNPs-based food packaging systems.

## 8 Future perspectives

To fully realize the potential of LNPs in food packaging, future research and development efforts must adopt a multidisciplinary, systems-level approach that addresses existing bottlenecks while opening new avenues for innovation. One of the most urgent priorities is the standardization of lignin feedstocks and extraction methodologies. Establishing a set of reference lignin types, based on plant origin (e.g., hardwood vs. softwood), extraction method (kraft, organosolv, DES), and functional group profile, would enable more consistent nanoparticle synthesis and facilitate inter-laboratory reproducibility. Coupled with this, predictive modeling and machine learning approaches could be employed to relate lignin structural parameters to nanoparticle morphology and film performance, streamlining the design process.

In parallel, the field must accelerate the development of next-generation LNPs through chemical and biological engineering strategies. These include grafting polymer chains (e.g., PLA, PEG, polycaprolactone) onto LNPs surfaces to improve dispersion and interfacial adhesion, and using enzyme-mediated polymerization (e.g., laccase-assisted coupling) to create more reactive or responsive nanoparticles. Recent work with hollow and porous LNPs, hybrid structures incorporating metal oxides (e.g., ZnO–LNPs), or multi-layered nanocapsules suggests exciting possibilities for embedding controlled-release functions, such as gradual antimicrobial agent diffusion or pH-triggered flavor enhancement. Additionally, electrospray and microfluidics-based synthesis platforms offer scalable, solvent-minimized routes to produce monodisperse LNPs with tailored morphology and surface functionality, especially relevant for industrial coating and lamination processes.

Beyond materials engineering, a promising direction lies in the expansion of substrate systems. Moving past conventional matrices like PLA or chitosan, newer biopolymer platforms such as marine polysaccharides (e.g., alginate, carrageenan), bacterial cellulose, PHBV, or macroalgae-derived films can offer regionally available, underutilized feedstocks with unique barrier or sensory properties. Integrating LNPs into these matrices may enable fully biobased, compostable, and edible packaging systems with competitive functional profiles. Moreover, layer-by-layer (LbL) assembly, spray-coating, and *in situ* nanoparticle generation within biopolymer films present flexible and decentralized fabrication strategies suitable for post-harvest or retail-level packaging customization.

A crucial parallel track involves regulatory alignment and risk assessment. Future studies must systematically address LNPs migration dynamics, biodegradation pathways, and ecotoxicological impact across various packaging scenarios, including acidic or fatty foods, UV-exposed conditions, and high-humidity storage. This requires standardized testing protocols, longer-term stability studies, and mechanistic insight into how nanoparticle structure influences release kinetics or degradation byproducts. Collaborative engagement with food safety agencies will be key to streamlining approval processes for LNPs-integrated packaging.

Finally, the life cycle analysis (LCA) and techno-economic assessment (TEA) of LNPs-based packaging systems must be integrated early in the design pipeline. These tools can guide material selection, process optimization, and commercial positioning by quantifying carbon footprint, water usage, and end-of-life impacts compared to traditional petroleum-based alternatives. In parallel, developing modular, open-source synthesis and coating toolkits for small-scale producers (e.g., in agri-food cooperatives) could democratize access to LNPs technologies and facilitate adoption in decentralized, low-waste packaging ecosystems. Thus, the future of LNPs-based food packaging rests on a convergence of materials science, green chemistry, regulatory science, and industrial engineering. By addressing current limitations and embracing innovations in lignin valorization, nanoparticle synthesis, and biopolymer integration, LNPs have the potential to become core components in high-performance, sustainable, and safe food packaging solutions.

## 9 Conclusion

Lignin nanoparticles represent a transformative opportunity to bridge the gap between environmental sustainability and functional performance in food packaging. Their integration into biodegradable polymers not only addresses key material limitations, such as poor mechanical properties, UV degradation, and lack of bioactivity, but also valorizes a largely underused biomass byproduct. Through careful control of extraction methods, nanoparticle synthesis, and matrix compatibility, LNPs can impart multifunctional attributes including enhanced barrier properties, antimicrobial activity, and photoprotection. Moreover, their inclusion aligns with circular economy goals by promoting resource efficiency, reducing reliance on petroleum-derived plastics, and supporting compostable or recyclable packaging formats. However, to fully realize their potential, critical challenges must be addressed, including nanoparticle uniformity, long-term stability, regulatory compliance, and end-of-life behavior. With growing innovation in nanoparticle engineering, hybrid design strategies, and substrate diversification, LNPs-based packaging systems are poised to become integral to the next generation of sustainable food contact materials. This review underscores the need for integrated approaches that combine materials science, green chemistry, and industrial feasibility to bring these promising systems into widespread use.
